# User Models for Personalized Physical Activity Interventions: Scoping Review

**DOI:** 10.2196/11098

**Published:** 2019-01-16

**Authors:** Suparna Ghanvatkar, Atreyi Kankanhalli, Vaibhav Rajan

**Affiliations:** 1 Department of Information Systems and Analytics School of Computing National University of Singapore Singapore Singapore

**Keywords:** review, exercise, physical fitness, automation, mobile apps, web browser, health communication, health promotion

## Abstract

**Background:**

Fitness devices have spurred the development of apps that aim to motivate users, through interventions, to increase their physical activity (PA). Personalization in the interventions is essential as the target users are diverse with respect to their activity levels, requirements, preferences, and behavior.

**Objective:**

This review aimed to (1) identify different kinds of personalization in interventions for promoting PA among any type of user group, (2) identify user models used for providing personalization, and (3) identify gaps in the current literature and suggest future research directions.

**Methods:**

A scoping review was undertaken by searching the databases PsycINFO, PubMed, Scopus, and Web of Science. The main inclusion criteria were (1) studies that aimed to promote PA; (2) studies that had personalization, with the intention of promoting PA through technology-based interventions; and (3) studies that described user models for personalization.

**Results:**

The literature search resulted in 49 eligible studies. Of these, 67% (33/49) studies focused solely on increasing PA, whereas the remaining studies had other objectives, such as maintaining healthy lifestyle (8 studies), weight loss management (6 studies), and rehabilitation (2 studies). The reviewed studies provide personalization in 6 categories: goal recommendation, activity recommendation, fitness partner recommendation, educational content, motivational content, and intervention timing. With respect to the mode of generation, interventions were found to be semiautomated or automatic. Of these, the automatic interventions were either knowledge-based or data-driven or both. User models in the studies were constructed with parameters from 5 categories: PA profile, demographics, medical data, behavior change technique (BCT) parameters, and contextual information. Only 27 of the eligible studies evaluated the interventions for improvement in PA, and 16 of these concluded that the interventions to increase PA are more effective when they are personalized.

**Conclusions:**

This review investigates personalization in the form of recommendations or feedback for increasing PA. On the basis of the review and gaps identified, research directions for improving the efficacy of personalized interventions are proposed. First, data-driven prediction techniques can facilitate effective personalization. Second, use of BCTs in automated interventions, and in combination with PA guidelines, are yet to be explored, and preliminary studies in this direction are promising. Third, systems with automated interventions also need to be suitably adapted to serve specific needs of patients with clinical conditions. Fourth, previous user models focus on single metric evaluations of PA instead of a potentially more effective, holistic, and multidimensional view. Fifth, with the widespread adoption of activity monitoring devices and mobile phones, personalized and dynamic user models can be created using available user data, including users’ social profile. Finally, the long-term effects of such interventions as well as the technology medium used for the interventions need to be evaluated rigorously.

## Introduction

### Background

Insufficient physical activity (PA) is a worldwide concern as it is a major cause of obesity and the fourth leading risk factor for mortality, accounting for an estimated 3.2 million deaths globally [1]. Maintaining or increasing PA of patients is also an important goal in the treatment for various chronic diseases such as diabetes and cardiovascular illnesses.

Fitness trackers, such as Fitbit and Jawbone, are increasingly being used to monitor personal PA. Activity data collected by associated smartphone apps are being utilized, along with other user-specific or contextual data, to design interventions with the aim of motivating users to increase their PA [2,3]. These interventions take varied forms ranging from activity status reports to personalized fitness-buddy recommendations.

Increasing PA often requires a change in lifestyle or behavior of the user. Feedback based on activity status reports is a common strategy that is often augmented with educational information on the benefits of increased PA. The key limitation of such interventions is the reliance on the self-motivation of users to increase their PA [4,5]. Users may not be motivated for various reasons, for example, they may be inactive by habit or the presented activity goal may be too intimidating for them. Other factors may also play a role in determining the efficacy of interventions. For example, some users may not have the time to perform a recommended PA [6] or there may be constraints imposed by the users’ location, weather, or working environments. Providing information on the benefits of increased PA rarely suffices; effecting behavior change to increase PA additionally requires motivational interventions [7].

The aims, behavior, preferences, context, and lifestyle of users have to be taken into account by apps to design effective interventions [8,9]. A “one size fits all” approach is unable to effectively serve a diverse set of users. Even simple activity recommendations, such as “60 minutes of moderately vigorous physical activity (MVPA)” may be too daunting for a sedentary user or for a cardiac patient. Thus, there is a need for personalization of interventions for promoting PA among users. Personalization implies a modification in the intervention generation or delivery aimed at a specific user. A status feedback does not indicate personalization; personalization implies customized content or advice to help the targeted user in increasing PA.

Previous reviews [10-13] on interventions for increasing PA have studied internet-based or Web-based interventions without focusing exclusively on personalization. They evaluated the success of included studies with respect to intervention delivery (eg, email and website-based) and discussed the utility of theory-based interventions [10]. Other reviews have specifically studied target groups, such as stroke patients [14] or cardiovascular disease (CVD) patients [15]. Another recent review [16] has analyzed the decision support systems used in PA interventions but does not focus on personalization. A survey of tailoring techniques used in real-time PA coaching systems published before August 2013 is presented in the study by op den Akker et al [17].

The term “personalization” has multiple definitions in different domains [18]. We follow the commonly accepted definition in the study by Fan et al [18], which defines personalization as “a process which changes the functionality, interface, content or distinctiveness of system to increase its personal relevance.” According to this definition, if the system is not altered in any of the dimensions mentioned to increase personal relevance, it is not considered as personalization. An earlier study by Hawkins et al [19], defines “tailoring” as a generic term for providing feedback, personalization, and content matching. It uses the term personalization to encompass the tactics of identification, raising expectation, and contextualization. However, following our adopted definition, from the study by Fan et al [18], we also include the category of “content matching” within “personalization.” The review by Akker et al [17] identified 7 categories based on tailoring techniques for activity coaching—feedback, inter-human interaction, adaptation, user targeting, goal setting, context awareness, and self-learning—and discussed relevant studies in these categories. Thus, tailoring has been used as a broad term in the literature and does not necessarily provide the “modification” required for personalization in our adopted definition. In this study, we use the term “personalization” to denote a user-specific modification of an intervention.

### Objectives

The purpose of this review was to identify recent literature where the technology-based intervention is personalized with the aim of increasing PA of users. The feedback or recommendation is not just a presentation of the users’ activity status. It is either a personalized feedback based on the history and status of the user to motivate or educate the user or a recommendation to potentially increase PA. A key aspect of such studies, which we focus on, is the user model created, which in turn helps generate personalized recommendations. Findings from this review provide important insights into the current literature and identify significant gaps in the literature. Addressing these gaps could lead to more effective, personalized, and technology-based interventions for promoting PA.

## Methods

### The Scoping Review

This review aims to identify various interventions, customizations, and user models generated for personalization of technology-based interventions to increase PA of users. We employed a rigorous literature search and chose to conduct a scoping review to analyze our research questions. The research questions we have focused on are as follows: (1) what are the ways of providing technology-based personalized interventions for increasing PA among users and (2) what are the user models used to provide such personalization? For ensuring quality of the included studies, we have used only peer-reviewed articles, including research-in-progress articles, which had full text available. We do not perform additional quality analysis of the studies, as quality assessment does not form part of the scoping study remit [20]. This paper follows the methodology and directions given in the study by Arksey and O’Malley [20].

### Search Strategy

PubMed, PsycINFO, Scopus, and Web of Science databases were used to select relevant studies. A comprehensive search was conducted till August 23, 2018, in which articles published since 2013 were targeted. The search string was constructed by considering the criteria required to be satisfied by the studies to be considered: {physical activity} {interventions} having {personalization} provided through some {technology} and identifying or creating a {user model} for the same. The following search string was used: ((fitness OR exercise OR “physical activity” OR “activity level” OR “active living”) AND (intervention OR recommend* OR prescribe OR prescription OR feedback OR message) AND (tailor* OR personaliz* OR personalis*) AND (mobile OR internet OR computer OR device OR “fitness trackers” OR website OR online) AND (profil* OR model)). The search was restricted to papers published in English. This search string ensured our condition of the technology-based intervention having a user model or profile identified for providing the personalization.

In addition to the database searches, we also performed hand searches for additional relevant studies. These studies were found by identifying relevant references from the studies selected. These references were also analyzed for the selection criteria and included in the review if they met the criteria. In addition, a hand search of *Journal of Medical Internet Research* results for “physical activity interventions” was done to identify several other relevant studies.

### Data Extraction

We selected the articles in 2 phases and used Mendeley reference manager to organize them. The first phase involved title, abstract, and keyword review as obtained from the databases searched. This phase was applied to all results obtained from the databases after merging duplicates (a feature provided by Mendeley). The second phase included reviewing the full text of the articles. This was done by obtaining PDF documents for each of the articles that met the inclusion criteria. The full texts were analyzed using the inclusion and exclusion criteria, and studies that were deemed relevant after this phase were included in the scoping review.

### Selection Criteria

Studies were eligible for this review if all the following were true: (1) there was an attempt to increase or regulate PA among the target users; (2) the studies had some form of personalized intervention, as recommendations or feedback intended to promote PA of the users; (3) a user model was generated and used for providing the personalized intervention described; (4) the intervention was provided through usage of technology; (5) studies were in English and published in or after the year 2013; and (6) they were not review papers, dissertations, or letters and were published through a peer-reviewed process.

Studies published before 2013 were not included as the popularity of fitness devices and attempts to create trackers and coaches have increased in the last 4 to 5 years, that is, older literature may be less relevant to today’s apps. Moreover, relevant literature until then has already been reviewed in the study by Akker et al [17]. There was no restriction on the study objectives, type of users, or the type of intervention or feedback, other than the focus on personalization for PA promotion. The focus of the review is on methods of personalization and user model generation for technology-based interventions. The interventions where personalization was provided manually were excluded as the user model used for delivering the personalization cannot be identified in a manual process. A comprehensive review of different ways to model users and provide personalized, technology-based interventions for increasing PA in different settings was desired.

The exclusion criteria for this review were as follows: (1) personalization not aimed at increasing PA (eg, personalization in activity tracking or gait detection); (2) personalization provided only in terms of using name or activity status in message, these parameters were filled into standard messages; (3) no user model identified during the intervention; (4) personalization generated manually, even though may be delivered using technology through a website; (5) gender- or culture-based standard tailoring for intervention; and (6) only reports provided without any personalized content for encouraging or educating user, or without any advice.

The inclusion criterion entailed that the technology-based, personalized intervention had to be necessarily aimed at increasing PA. The criterion of increasing PA was not necessarily the main objective of included studies but had to be one of the objectives. For example, in some studies, medication adherence [21] or weight loss [2] was the other objective.

## Results

### Screening and Study Selection

The screening procedure and study selection was undertaken by 1 researcher and then independently verified by 2 other researchers for adherence to the selection criteria. The initial results were screened for the inclusion criteria and the full-text articles were analyzed using the exclusion criteria. Initial results were obtained by setting the filters of language and duration for all the databases (536 results) and were then searched for duplicates, which resulted in 355 unique studies. The abstracts of these studies were then screened for the criteria of whether the paper tried to increase or regulate PA. In addition, 15 relevant studies were identified by hand searching and cross-references. This led to a selection of 181 papers, which were screened on full text for the remaining selection criteria, resulting in 57 studies.

We found several groups of studies that studied the same system, that is, they were parts or improvements of the same intervention. We also found additional studies through hand searches that belonged to these groups, which helped us understand details of the interventions. We grouped these related studies together and used only 1 representative publication for each of the 23 groups. The groups and the representative studies are listed in Table 1. This step reduced the final number of studies to 49.

**Table 1:** Studies grouped by the intervention developed or investigated.

Intervention	Related studies	Representative study
ACKTUS	Janols et al and Lindgren et al [23,24]	Janols et al [23]
Active Plus	Boekhout et al, Peels et al, Peels et al, and van Stralen et al [25-28]	Peels et al [27]
Active2Gether	Klein et al and Klein et al [3,29]	Klein et al 2017 [3]
Active-O-Meter	Cook et al and De Bourdeaudhuji et al [30,31]	Cook et al [30]
ATHENA	Ali et al and Fahim et al [32,33]	Fahim et al [32]
Food4Me	Marsaux et al, Morales et al, and Marsaux et al [34-36]	Marsaux et al [35]
I Move	Friederichs et al and Friederichs et al [37,38]	Friederichs et al [38]
MOPO	Ahola et al, Jauho et al, and Pyky et al [39-41]	Pyky et al [41]
My Activity Coach	Alley et al and Alley et al [42,43]	Alley et al [43]
MyBehavior	Rabbi et al [44,45]	Rabbi et al [45]
myHealthyBehavior	Schulz et al and Schulz et al [46,47]	Schulz et al [47]
PATH-In	Brooks et al and Williams et al [48,49]	Williams et al [48]
PATHway	Chatzitofis et al, Claes et al, and Triantafyllidis et al [50-52]	Triantafyllidis et al [52]
Personalized Coaching System	Cabrita et al, Hermens et al, and Op den Akker et al [53-55]	Hermens et al [54]
PRO-Fit	Dharia et al [56-58]	Dharia et al [56]
REACH	Mitchell et al and Mitchell et al [59,60]	Mitchell et al [60]
RENATA	Reinwad et al and Storm et al [7,61]	Storm et al [7]
SmartLoss	Martin et al and Martin et al [2,62]	Martin et al [2]
Start to Stand	De Cocker and De Cocker [63,64]	De Cocker [64]
TaylorActive	Soetens et al and Vandelanotte et al [65,66]	Vandelanotte et al [66]
TXT2Bfit	Hebden et al and Partridge et al [67,68]	Partridge et al [68]
Weight in Balance	Walthouwer et al, Walthouwer et al, and Walthouwer et al [69-71]	Walthouwer et al [71]
YEAH	Kattelmann et al [72,73]	Kattelmann et al [72]

Among the 181 studies assessed for eligibility, most of the studies could be screened using our exclusion criteria. However, a few studies, such as the study by Liu and Chan [22], were identified through the search but were excluded because the definition of personalization used was different. It focused on whether or not to prompt the user based on current and predicted activity status, which differs from the conceptualization adopted.

**Figure 1:**
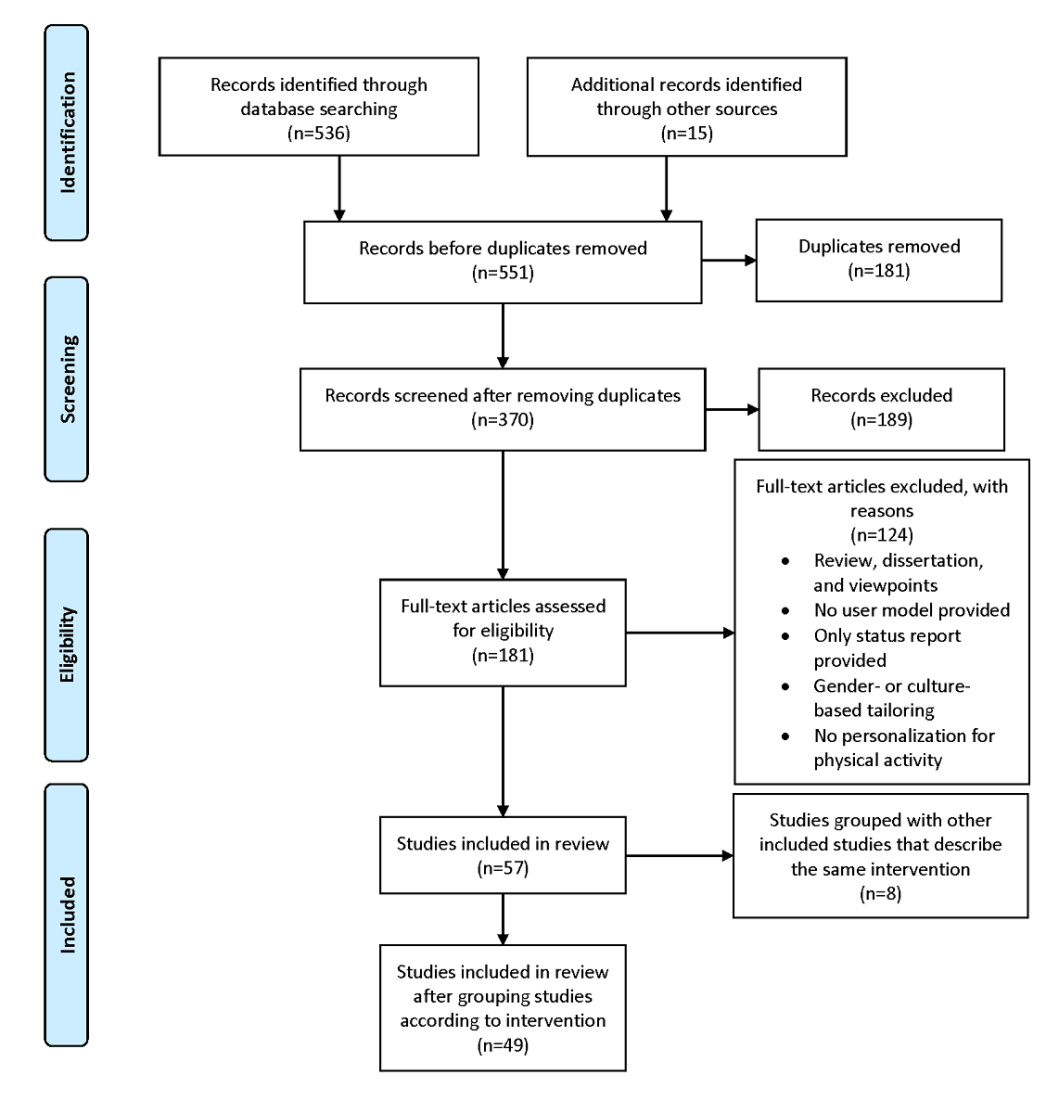
Flowchart for study selection process.

Figure 1 illustrates the flowchart representing the study selection process.

### Overview of Studies

We placed no restrictions on the research objective or methodology of the studies to be included in the review, other than following our study criteria. As a result, the studies differ considerably with respect to their research objectives, interventions, data collection methods, and target users. We summarize these diverse settings below before examining the personalized interventions and user models employed in more detail. Moreover, as mentioned earlier, we identified 23 interventions, each of which has been described in more than 1 study. These groups are listed in Table 1. In this review, we study all the articles listed in the table (column 2) but represent each group by a representative study (column 3).

Increasing PA was the research objective in 33 studies. In the remaining studies, the objectives were weight loss, weight management, or obesity prevention (6 studies); maintaining healthy lifestyle that included diet, smoking, alcohol, or exercise management (8 studies); and rehabilitation (2 studies), with PA increase being an auxiliary goal. Furthermore, 1 study had a combined goal of weight management and healthy lifestyle. Among the 33 studies on PA, 31 directly aimed to increase PA of users, 1 study aimed to reduce workplace sitting time [64], and the last study aimed to encourage medical adherence in addition to increasing PA [21]. These studies not only tried to monitor and increase PA but some also focused on helping users overcome barriers to increasing PA and improving their self-efficacy, for example in Oosterom-Calo et al [21]. The 6 studies aimed at weight loss attempted to increase the PA of users to achieve the desired results [eg, 2,74]. One study aimed at providing rehabilitation to patients provided recommendations in consultation with health care experts [75]. The last study aimed at rehabilitation provided real-time as well as weekly adaptation of exercises for the patients [52]. For the 8 studies on healthy lifestyle, their objectives included wellness services [32], exercise and diet recommendation [6,7,47,76-78], and a personalized coaching system [54], which was illustrated through 3 use cases, that is, neck coach, activity coach, and stress coach.

The interventions were presented or delivered to users in multiple ways, for example, through Web apps [7,66,79], mobile phone apps [2,3,74], Kinect devices [52,80], specific activity monitors [81], PDF report [35], text message (short message service, SMS) [9,68,82], printout [27,83,84], or telephone call [60]. In some cases the users were actively pushed by the system toward their PA goal by automatic delivery of interventions periodically [7,9,54]. In other cases, the interventions were relatively more passive and expected higher levels of motivation from the users. They required the user to access the app [45] or answer questionnaires [21,64,79] before they could obtain the personalized intervention.

Data were collected for the intervention (for monitoring users and generating user models) in various ways, for example, through questionnaires [3,21,80], mobile phone sensors [9,32,45,85], specific activity monitors [41,64,66,81], or fitness trackers [2,3,86]. The target population of the studies varied from specific to general users, that is, people with chronic disease [76], elderly adults [79,80], diabetes patients [9,87], cancer patients [78,82,84], people with CVD or at risk of CVD or heart failure [7,21], osteoarthritis patients [48], young adults [3,72,88], and general users [2,32,45,86].

Apart from these differences, the included studies differed in the intervention generation techniques, type of personalization, and user models. In this review, we systematically study these 3 aspects in detail. Note that some studies included in the review may also have incorporated personalization of diet or models for activity detection, but our focus is restricted to only the part of the study concerning PA. The overview presented in Multimedia Appendix 1 highlights the objectives, interventions, personalization, user models, and theoretical models used in the included studies. These are ordered by their research objectives and by the intervention generation mode within each objective.

### Types of Interventions

The interventions included in this review provide various forms of feedback or recommendation. We distinguish between feedback and recommendation, where recommendations are prescriptive in nature, whereas feedback is an informative response to the users’ actions. For example, feedback can be information regarding tips to increase PA such as “exercising with a partner can be a fun and motivating experience,” whereas recommendations are prescriptive suggestions of an activity or goal such as “30 minutes of brisk walk along with your mother” provided to the user. We only consider feedback that is personalized in some aspect (eg, content or timing).

Personalization is achieved in different ways, that is, by personalizing goal, activity, or fitness partner recommendations or by personalizing messages and their timings, as discussed in the following section. On the basis of how this personalized intervention is generated, interventions in our review could be classified into 2 categories: semiautomated and automated. We excluded those studies that had only manual interventions.

#### Semiautomated Interventions

Semiautomated interventions are those where personalization is not completely automated but includes manual effort from the health care provider. There were 9 studies with semiautomated interventions, and the combinations of manual and automatic elements in them varied.

In the study by Tseng et al [76], automated activity and goal recommendations were provided, which could be modified by a medical expert. Similarly, for the case of SmartLoss [2], the system required a goal to be set in consultation with the nurse, but the platform also automatically provided a set of “SmartTips” in case the user was predicted to be deviating from the weight loss program. Another semiautomated system [35] provided automated educational content to the users using a machine learning method along with manual personalized advice and intervention from an expert. My Activity Coach study [43] used automatic advice recommendation as well as a one-on-one video calling interaction with coach. Similarly, 3 other studies [60,68,82] used telephonic conversations for motivational interviewing, but the participants had website access or automatically delivered messages. The remaining 2 studies [75,80] generated automatic personalized activity or game levels within the limits defined by a health care expert.

#### Automated Interventions

Automated interventions present in 40 papers in our review used either knowledge-based or data-driven approaches or both to automate the personalization. All the knowledge-based systems relied on either behavior change techniques (BCTs) or PA or clinical guidelines. All the data-driven systems used machine learning techniques to learn user models from historical data.

##### Knowledge-Based Systems

There were 30 studies using knowledge-based approaches. These systems were rule-based and provided feedback and recommendations based on reasoning modules or rules.

Of these studies, 22 of them attempted to encode knowledge into the system derived from behavior change theories. They provided the personalization intervention by inferring the most suitable user category, where the categories were theory-based. Thus, they provided category-level personalization instead of an individual level of personalization. In addition, 2 studies added an individual level personalization by considering the preference of intervention time [89,90] or by providing walking and cycling routes based on location [27].

There were 8 studies that used PA or clinical guidelines. The study by Ali et al [85] used a hybrid rule- and case-based reasoning model but tried to identify similar cases using the K-nearest neighbor algorithm. Their system was based on the Center for Disease Control and Prevention (CDC) guidelines [91]. The study by Coolbaugh et al [81] used a specific activity monitor for providing the intervention and performed goal adaptation in accordance with American College of Sports Medicine training progression guidelines [92]. This intervention was time-bound and progressed according to the rules laid out in the process flowchart. Moreover, 2 studies [48,93] also used various PA guidelines to recommend step goals. The last 3 studies [52,78,87] used PA guidelines for their specific types of users, that is, cancer patients and diabetes patients. Few studies among these [6,52,78,87] use knowledge-based system constructed using a BCT but provide recommendations based on clinical guidelines.

##### Data-Driven Systems

Data-driven systems using machine learning approaches were described in 7 studies. The class of machine learning techniques used falls under 2 broad categories, that is, reinforcement learning [9,45] and supervised learning [35,56,74,86,94].

In reinforcement learning, an automated agent learns a policy to optimize a cumulative reward function while sequentially interacting with the environment. At each step, the agent performs an action, obtains a reward, and decides its next action based on the reward with the aim of optimizing the reward function. Thus, in the study by Yom-Tov et al [9], at each step, the agent sent a message, obtained information about the user’s PA, and determined which message to send in the next step. The messaging policy was personalized for the user to maximize cumulative PA. For the other study, multi-armed bandits, a form of reinforcement learning, was used for suggestion generation in the MyBehavior app [45].

Supervised machine learning techniques learn a model from historical data to predict dependent variables from independent variables. The model may be static (such as support vector machine [SVM] or decision trees in the study Marsaux et al [35]) or temporal (such as recurrent neural networks [RNN] in the study by Lim et al [86])—the former does not explicitly model temporal correlations, whereas the latter does. In another study, PRO-fit recommended a fitness partner using geolocation, activity preference, and calendar-based availability on a smartphone [56]. It also provided activity recommendation using collaborative filtering [57] and activity prediction from raw accelerometer data. An Internet of Things–based app [94] proposed a context-aware recommendation system to generate a suitable activity for the user based on current fatigue and fitness level. Finally, in the study by Lim et al [86], a lifestyle model parameterized by heart rate (HR), step count, and burned calories was constructed using RNN, and recommendations for healthy behavior were based on forecasts of these variables.

##### Combined Knowledge-Based and Data-Driven System

There were 3 studies that combined knowledge-based and data-driven approaches. The ATHENA system [32] defined a rule-based recommendation, in which only ranking and validation were done using machine learning. Ensemble-based supervised learning was used for recommendation of food, physical, and mental therapy in study. In the study by Hermens et al [54], a rule-based system was used for message content along with machine learning to appropriately time the message—an SVM was trained based on historical data to predict effective timing. Another personalized health care system [77] proposed an ontology-based knowledge base, which uses decision trees for providing relevant recommendation to the user.

### Overview of Personalization

Personalization was found in both recommendations and feedback. In the case of recommendations, personalization was seen with respect to goal setting, activity suggestion, and selection of fitness partners. Feedback was found to be personalized with respect to the content, which could be motivational or educational, or with respect to the timing of its delivery. Status comparative feedback was also considered to be personalized as it was provided to only those for whom it was considered beneficial. Thus, we classify personalized interventions into 6 categories, that is, goal recommendation, activity recommendation, fitness partner recommendation, educational content, motivational content, and intervention timing. These categories are not mutually exclusive, as several studies had more than 1 type of personalization.

#### Goal Recommendation

The category of goal recommendation refers to the prescription of a quantified target goal. This target is in terms of an activity evaluation metric, such as duration of activity, step count, or calorie expenditure. Note that if an activity is prescribed without quantification, then we classify it as an activity recommendation and not a goal recommendation.

Of the studies in the review, 20 of them provided personalized goal recommendation. The specification of the goals differed across the systems and apps. The goals could be specified in terms of game level [80], training zone and HR [52,75,81], activity duration [3,43,76,81,82,89], step data [2,54,60,93,95], or activity level prescription by an expert [23]. These goals were adapted according to the person’s status and did not follow standardized fixed goals (such as “30 minutes of MVPA”). For the case of the REACH intervention [60], it has been mentioned that personalized step goals are generated based on rate of perceived exertion. However, it is not stated if this is done automatically or by researchers and delivered manually.

In 4 of these studies [2,45,66,85], personalized goals were indirectly defined or altered after obtaining fixed goals from the user or a guideline. The SmartLoss app [2] aimed to make the user follow their regular exercise program of 7000 to 8000 steps per day. It defined a “zone of adherence,” which is a weight range indicating that the weight loss of the user is as expected. Goal adaptation occurred when a user was repeatedly outside this “zone of adherence” and was provided other options for increasing PA. In the multimodal reasoning system [85], the example goal was in terms of kilograms to lose, but the personalized goals in terms of target metabolic equivalents (METs) and calories were also calculated and recommended by the system. The MyBehavior app [45] used the weekly weight loss goal entered by the user to obtain a personalized target calorie goal using the Harris-Benedict equation [96]. The TaylorActive system [66] also provided goal recommendations and suggestions during a session, but the actual goal was set by the user.

All the above-mentioned studies set a goal for the user before the user activity began. However, in the personalized PA prescription intervention study [81], the goal was not explicitly known by the user before the activity, although a Web interface allowed the user to check the goal recommendation. It also defined a user goal in terms of target HR and duration of activity, which was sent to the activity monitor. The activity monitor provided visual feedback (blinks on the monitor) to the user when the target goal was achieved or if their HR exceeded the target.

In another study [35], the feedback was whether the user must increase, strongly increase, or maintain their PA. However, the feedback was not quantified, and thus, this study was not classified as a goal recommendation.

#### Activity Recommendation

The category of activity recommendation includes studies where 1 or more appropriate activities (eg, running and cycling) or behaviors (eg, sleep for X hours) are prescribed to the user. The 22 studies in our review that provided activity recommendation also retained the monitored PA as part of the user model.

Of these, 4 studies offered semiautomated interventions. The activity recommendations were in the form of health care experts’ treatment advice (where the treatment included PA) [80] or activity suggestions [35,76]. Furthermore, 1 study [82] did not use a health care expert for the activity plan but suggested activity in the messages sent to the user.

The remaining 18 studies generated activity recommendations based on automated systems. These studies generated the activity or behavior recommendation by considering contextual information such as location [45,56,86] or preferences [32,66,85].

In another study [64], the recommended activities were restricted to standing or walking. It is also important to note that this system encouraged the user to create a goal and activity plan with the aid of the system, which shows the importance of user’s motivation and involvement in planning. In the study by Klein et al [3], the activity and goal recommendations were provided, but the final choice was left to the user. Similarly, in the study by Williams et al [48], the activity plan was generated by the system and adjusted to user level, but the user could rerequest the plan generation. For the personalized coaching system in the study by Hermens et al [54], the activity recommendation was provided in the content of the message [54].

#### Fitness Partner Recommendation

The aim of fitness partner recommendation is to match users of a system who are similar, to motivate them and help them maintain their PA. Of the 3 studies of this type, 2 studies [56,74] used recommender systems for finding a suitable partner. The other study [95] attempted to find a similar user by matching all users who crossed the particular user during a running activity.

#### Educational Content

In the educational content category (21 studies), personalized feedback aimed to increase the knowledge of the users about the importance of or techniques for improving PA.

There is a vast amount of information available on the internet, and providing the user with the most relevant content is the aim of such personalization. A direct way to do this was to provide appropriate links to website content (eg, in Food4Me [35], SmartLoss [2], and the multimodal system in the study by Ali et al [85]). The “My Activity Coach” system [43] and Ninas Saludables intervention [88] provide tailored Web content to users, for example, obese users receive additional content not provided to users with normal weight. The Active2Gether system [3] had an educational phase, where messages that put a user’s insufficient performance into perspective were sent along with the need and benefits of PA. The “start to stand” [64] app provided feedback messages, which also helped impress the harmful effects of too much sitting or sedentary lifestyle, based on the decision rules. Some studies such as those by Storm et al and Short et al [7,84] provided tailored example plans to the user to aid in goal setting. Some studies provided content in terms of tips to increase PA if found to be relevant [30,38,71,97,98]. The I-Move for Life study [84] provided information on the benefits of PA tailored according to the expected outcomes. The Active Plus system [27] provided the user with information on sports opportunities tailored to the location, along with walking and cycling routes. This information is also educational as it provides a feasible method for improving PA of the user.

Educational content may be motivational as well, for example, if the content is provided to help users overcome their specific barriers to performing PA [21,32,66,79,83]. This was in the form of tailored video or textual content.

#### Motivational Content

This category (29 studies) contains personalized feedback that aims to motivate users to improve their PA. What may motivate a user can be inferred from specific rules or BCTs. Note that motivational messages that were not personalized (eg, “Good!” [2]) were not included. Messages in this category targeted users specifically to elicit an action by also utilizing techniques including the users’ name or providing users’ current PA status [3]. However, as mentioned in the exclusion criteria, using only statistics or name in a standard template message is not considered personalization.

A reinforcement learning based study [9] aimed to learn which type of message (negative feedback, positive relative to self, positive relative to others, or no message) best motivates a user. A few studies had both motivational and educational messages as they target the beliefs of users [43,83,84,88]. The studies targeting “stage of change” of the user generally provided personalized motivation [3,41,54,68,72] by determining the stage the user is currently in, for example, precontemplation, preparation, or maintenance. The TaylorActive system [66] provided personally relevant feedback in various categories including what they called the “boosting your confidence” category.

The multimodal system in the study by Ali et al [85] offered motivating content that was not personalized. The Social POD app [74] provided personalized fitness partner recommendation. Personalization of motivational content was done through the fitness partner, who selected a motivational message to be sent to the user.

#### Intervention Timing

This form of personalization takes the context into account and finds the right time to send a feedback or recommendation to the user. Timing of feedback is known to play an important role, for example, a notification reminder sent when the user is busy is likely to be ignored and forgotten.

In our review, 7 studies provided this kind of personalization. Of these, 2 studies [54,86] learned the most appropriate time for intervention from past data using machine learning. The neural network–based model [86] used a greedy policy to determine the best time, which learns from user feedback, after predicting the users’ activity. The personalized coaching system in the study by Hermens et al [54] trained an SVM to determine the appropriateness of a given time to send a message. Moreover, 2 studies (PRO-Fit [56] and Step Up Life [89]) used the calendar context to determine if a given time was suitable for recommendation. Step Up Life intervention [89] additionally used location to determine home and “friendly” locations for providing intervention or reminders. Furthermore, 2 studies [82,90] used the preference of timing obtained from user for providing personalization. The last study [6] mentioned sending reminders at opportune moments, but the exact methodology is unclear.

#### Theoretical Background for Personalization

In our review, 41 studies used a theoretical framework or foundation for providing personalization. Apps with a theoretical background for their personalization either followed guidelines from sports or health care bodies or used BCTs.

Activity training guidelines from the American College of Sports Medicine [92] were followed for recommending activity increments to avoid injuries in the studies by Lee et al and Coolbaugh et al [78,81]. Guidelines from the CDC [91] were used to generate hybrid rule-based techniques to recommend a suitable activity to users in the studies by Fahim et al and Ali et al [32,85].

BCTs are theory-based methods for changing 1 or several psychological determinants of behavior, such as a person’s attitude or self-efficacy. They aim to create a change among users through appropriate persuasion. Several studies used knowledge-based approaches to incorporate BCTs in providing personalization. The BCTs used were based on Fogg’s Behavior Model [99], Social Cognitive Theory (SCT) [100], Transtheoretical Model (TTM) [101], Theory of Planned Behavior (TPB) [102], I-Change Model [103], Behavioral Change Wheel [104], Activity Theory [104], Protection Motivation Theory [105], Motivational Interviewing [106], and health action process approach (HAPA) [107]. The study by Mukhtar [6] used Fogg’s Behavior Model to create what is termed as a “persuasion strategy” for the user, which takes into account motivation, ability, and trigger as parameters for appropriate recommendation. The Step Up Life intervention also utilizes the Fogg’s Behavior Theory for designing the model [89]. HAPA [107] was used to target different user stages and provide information on behavior risk and intention formation to the user in the study by Storm et al [7]. TTM defines stages of change in users and was used to determine the feedback given to the user, a direct rule-based implementation of the underlying BCT in the study by Pyky et al [41]. A system utilizing TPB and the Stage of Change Model [101] represented the constructs through questions as psychosocial correlates with PA. I-Change Model was used to design the system and questionnaires to effectively motivate users in a few studies [47,71,97]. The SCT has also been used to design the intervention considering that including and addressing social mediators such as family and peer would elicit a positive and sustained response from the user [60,88]. The TaylorActive study [66] used TPB, SCT, and self-determination theory to assess various constructs such as self-efficacy, intrinsic/extrinsic motivation, and action planning during different sessions designed for the user.

Theoretical frameworks were not present in studies using machine learning algorithms for recommending a goal or activity, as the algorithm was used to model user activity and suggest or recommend a better alternative. Some studies [45,74] used BCTs to make design decisions and choices but did not use BCT parameters for user modeling. For instance, the MyBehavior app [44,45] followed BCT to provide low-effort suggestions and used a form of reinforcement learning for activity recommendation. Another study [74] used SCT to design messages and used machine learning to recommend a fitness partner. Table 2 shows the different types personalization provided by the studies in our review.

### User Models

Each study in this review created a different user model and defined the user through various attributes. We classify user-related attributes into 5 categories, that is, PA profile, demographics, medical data, BCT parameters, and contextual information.

User models can have a static and/or a dynamic component. The static component is collected only once, typically at the start of the intervention, for example, demographics and preferences. The dynamic component gets updated regularly and includes the monitored quantity describing PA. Some user models also used the personalized quantity as part of the model. All the collected information may not be part of the user model; here, only the data required and used to provide personalization are described under the user model. In cases where it could not be determined how the measured quantity was used, it has been mentioned as part of the profile descriptions.

#### Physical Activity Profile

The user model nearly always included the quantity being monitored—weight, diet, or PA—either recorded automatically or logged by the user. The monitored quantity differs in the included studies because of differing research objectives, intervention systems, and evaluation metrics. PA profile consisted of this monitored quantity along with the historical data of feedback, goals, or activity.

Evaluation of PA was necessary in almost all cases as personalized advice to users would need to consider current PA status of the users. Thus, PA profile was used as part of the user model in 47 studies. However, PA profile data were not used in 2 studies that provided behavior advice to its users based on the assessment and identified problematic beliefs and barriers [21,68].

Most of the studies evaluated PA by calorie or energy expenditure in terms of METs [45,85,86]. Some others estimated it by the time spent at different PA levels, such as vigorous or MVPA [41,87]. There were studies which set the target HR and used specific HR monitors for data collection [80,81,95], whereas 1 used a smartwatch [75]. Step count was another measure used to evaluate PA, obtained directly from fitness tracking devices [2,3,9]. PA was also evaluated by the time spent in performing an activity [30,74] or the duration. Another study [35] used metrics such as PA level and activity energy expenditure to estimate the level and energy expenditure in performing the PA. Stairs climbed was also used as a measure of PA in the study by Klein et al [3]. The activity level was a common metric used by studies which collected PA-related data through questionnaires [38,43,79,97].

The parameters listed in the PA profile of the studies (see Multimedia Appendix 2) are self-explanatory except for 2 of them. The “start and stand” app [64] had a data attribute named “level of sitting time in 5 domains.” This was obtained through the Workforce Sitting Questionnaire and included time spent in (1) traveling, (2) at work, (3) watching television, (4) using computer at home, and (5) other leisure activities. In the study by Martin et al [2], the “zone of adherence” was a quantity calculated by their mathematical model to predict whether the user needs to be provided special interventions. Furthermore, 1 study [77] used the term “lifestyle” for personalizing the exercise recommendation to a person. This has been categorized as a PA profile metric as lifestyle can be used to deduce the current level of PA of the person. In addition, 1 study [60] used a metric termed as Signal Vector Magnitude to calculate the vector magnitude of acceleration corrected for gravity.

**Table 2:** Personalization provided.

Serial #	Paper reference	Goal recommendation	Activity recommendation	Fitness partner	Educational content	Motivational content	Intervention timing
1	Vandelanotte et al [66]	Y^a^	Y	—^b^	Y	Y	—
2	Ahire et al [77]	—	Y	—	—	—	—
3	Mukhtar [6]	U^c^	Y	—	—	—	Y
4	Tseng et al [76]	Y	Y	—	—	—	—
5	Storm et al [7]	—	—	—	Y	Y	—
6	Schulz et al [47]	—	—	—	—	Y	—
7	Hermens et al [54]	Y	Y	—	—	Y	Y
8	Lee et al [78]	Y	Y	—	Y	Y	—
9	Fahim et al [32]	—	Y	—	—	—	—
10	Dharia et al [56]	—	Y	Y	—	—	Y
11	Rabbi et al [45]	Y	Y	—	—	—	—
12	Twardowski et al [94]	Y	Y	—	—	—	—
13	Yom-Tov et al [9]	—	—	—	—	Y	—
14	Lim et al [86]	—	Y	—	—	—	Y
15	Cook et al [30]	—	—	—	Y	Y	—
16	Larsen et al [88]	—	—	—	Y	—	—
17	Short et al [84]	—	—	—	Y	Y	—
18	Boudreau et al [97]	—	—	—	Y	Y	—
19	Moreau et al [87]	—	—	—	Y	Y	—
20	Rajanna et al [89]	Y, U	Y	—	—	—	Y
21	Irvine et al [83]	—	Y	—	Y	U	—
22	Friederichs et al [38]	—	—	—	Y	Y	—
23	Blake et al [108]	—	—	—	—	Y	—
24	Coolbaugh et al [81]	Y	—	—	—	—	—
25	Hargreaves et al [93]	Y	—	—	—	Y, U	—
26	Williams et al [48]	Y-initially	Y	—	—	—	—
27	Kwasnicka et al [98]	—	—	—	Y	Y	—
28	Janols et al [23]	U	—	—	—	Y	—
29	Ali et al [85]	Y	Y	—	Y	—	—
30	Mistry et al [90]	—	—	—	—	Y	Y
31	Peels et al [27]	—	Y	—	Y	Y	—
32	Klein et al [3]	Y	Y	—	Y	Y	—
33	Ammann et al [79]	—	—	—	Y	Y	—
34	Pyky et al [41]	—	U	—	U	Y	—
35	Varadharajan et al [95]	Y	—	Y	—	Y	—
36	Codreanu et al [80]	Y	Y	—	—	—	—
37	Marsaux et al [35]	—	Y	—	Y	—	—
38	Alley et al [43]	Y	—	—	Y	Y	—
39	Mitchell et al [60]	Y, U	—	—	—	—	—
40	Oosterom-Calo et al [21]	—	—	—	Y	Y	—
41	De Cocker et al [64]	—	Y	—	Y	Y	—
42	Triantafyllidis et al [52]	Y	Y	—	—	Y (if required)	—
43	Dobrican et al [75]	Y	—	—	—	—	—
44	Hales et al [74]	—	—	Y	—	Indirect	—
45	Martin et al [2]	Y	—	—	Y	—	—
46	Spark et al [82]	Y, U	Y, U	—	—	Y	Y
47	Kattelmann et al [72]	U	U	—	—	Y	—
48	Partridge et al [68]	—	—	—	—	Y	—
49	Walthouwer et al [71]	—	—	—	Y	U	—

^a^Y: personalization present.

^b^Personalization absent.

^c^U: unclear.

#### Demographics

Demographics formed a part of the user model for 39 studies. Demographic data collected included age and gender [75], body mass index [7,32], employment [64,79], nationality [7], weight [79,95], marital status [47,88,97], and education [64,79]. Several studies collected demographic information but did not use it for providing the personalized intervention. User demographics formed an important part of the user model in 16 papers. Among these, some studies [7,41,54,74,79] did not explicitly state whether demographics was used for personalization or not.

#### Medical Data

In our review, 16 studies (aimed at rehabilitation, healthy lifestyle, and increasing PA) used medical data as part of their user model. Personalization was based on clinical symptoms [77,80], cholesterol levels [35,93], medical records [6,76,94], pain [23,48,52], and anaerobic threshold (the point between aerobic and anaerobic training of the user) along with HR and HR at rest [75]. It is unclear if the study by Mitchell et al [60] used the medical data for providing personalization. Sleep data were also collected by 2 studies [23,66]; however, whether it was directly used for providing personalization is not clear.

#### Behavior Change Technique Parameters

In our review, 30 studies used various BCT parameters such as stage of change [79]; subjective PA [3]; motivation [66]; skills, barriers, goals, and outcome expectations [3]; habit strength [7]; and rate of perceived exertion [60]. The MOPO study [41] based its personalization on a data attribute termed “life satisfaction,” which is a self-reported scale on happiness, interest in life, feeling of loneliness, and the ease of living. Various psychosocial parameters such as attitudes, intention, motivation, and confidence are also used along with stage of change [87,97].

Such BCT parameters were inferred using questionnaires such as the 20-item Weight Efficacy-Lifestyle Questionnaire and the 44-item Big Five Inventory Questionnaire that sought answers from users. Studies using BCT parameters had interventions that were knowledge-based, except in the studies by Hermens et al and Hales et al [54,74].

#### Contextual Information

Contextual information in the user models refers to any additional information that provided cues to the context and/or behavior of the user. The context of the user varied considerably across the 14 studies that used this type of information in our review. This category included user preferences, social media profile, location, time, mood, and energy levels among others.

Activity preferences of a user were generally obtained from the user to recommend a suitable activity to the user. All the 12 studies utilizing preferences also used PA profile in their user models. Preferences were also inferred based on users’ history and adherence to recommendations in the studies by Yom-Tov et al and Lim et al [9,86]. Location and time information was used to determine the feasibility of certain activity recommendations in the studies by Klein et al and Short et al [3,84]. For example, jogging may not be feasible during rainy weather. A study by Codreanu and Florea [80] used the estimated energy level (rested, fatigued, or energetic), defined by the “mood temperature factor.”

In our review, 4 papers used the social media profile to motivate users through activity status posts on websites or by inspiration from friends. Of these, 2 studies [32,95] used the profile to provide better recommendations and persuasion to users. On the other hand, the Active2Gether and PRO-Fit systems used social media in a direct way to generate social comparison [3] and recommend a fitness buddy to the user [56].

The TaylorActive app [66] used various indicators to gauge quality of life, perceived neighborhood environment, learning style, and delivery mode preference. All of these were measured using questionnaires provided to the user. Multimedia Appendix 2 summarizes the parameters across the 5 categories for the user models of the studies in our review.

### Results of Individual Studies

The studies included in this review have diverse aims and, thus, different evaluation metrics. For our review, we have considered only the results relevant to PA of users. Not all studies in the review presented evaluations of their proposed interventions and not all of them evaluated a PA metric. In our review, only 27 studies presented evaluations of their proposed interventions for PA. Out of these, 15 studies have reported positive statistically significant outcomes. The remaining 12 studies have not shown statistically significant results or shown no improvement at all. The impact and extent of the results vary in the studies as all are not randomized controlled trials (RCTs) and do not try to address similar questions. Table 3 shows the evaluation and results for the RCTs included in this review.

There are 20 RCTs (listed in Table 3), which evaluated their proposed interventions. Of these, some studies were evaluated on the basis of self-reported PA [7,47,83,84] and others used objectively measured PA through devices [35,41,66,93]. A metric used in these studies is MET-minutes or MET hours, which is the metabolic equivalent unit for energy expenditure. These MET minutes have been observed from self-reported data collected through questionnaires. We observe that not all studies report an improvement in PA after intervention as compared with the control group. In the study by De Cocker et al [64], the objectively measured sitting time has no significant difference; however, the self-reported data show significant difference between the intervention and control groups. The MOPO study [41] also reports significant change in self-rated fitness but no significant change in self-reported daily sitting time. On the other hand, studies such as the ones by Vandelanotte et al, Partridge et al, and Irvine et al [66,68,83] reported a significant improvement in PA of users after the intervention. The Reinforcement Learning(RL)-based messaging intervention [9] observed a significant improvement in the messages sent through the learned policy for the user in comparison with the initial random policy.

Some of the studies evaluated the difference in intervention delivery mediums. In the study by Peels et al [27], 2 kinds of personalized interventions were used—basic and environmental—where environmental intervention provided users with more contextual information, such as walking and cycling routes. In the study by Van Stralen et al [28], it was found that the printed interventions—basic as well as environmental—were significantly effective; however, the Web-based interventions were not. However, in the study by Walthouwer et al [71], no significant difference was observed when participants were provided interventions through the medium of their choice (text or video). Similarly, the study by Blake et al [108] observed no significant difference between delivery modes, email, and SMS. In addition, the study by Schulz et al [47] observed no statistical difference among the sequential intervention module delivery versus the simultaneous module delivery. Another study, the TaylorActive system [65], reported an increase in PA for all groups of intervention delivery—text, video, and combination.

Table 4 shows the evaluation of other studies (which are not RCTs), along with the methodology used for evaluation.

There are 7 studies in the review, which are not RCTs, but present some feasibility or usability analyses [74,81] or are observational [79,88] or single group studies [45,54,82]. Of these, some studies such as the studies by Hermens et al [54] and Coolbaugh et al [81] have very low sample size (8 and 2, respectively). The Personalized Coaching System study [54] conducted many different experiments. We consider the one mentioned in the paper, which aims to improve long-term activity behavior of chronic obstructive pulmonary disease patients. Moreover, 5 out of 8 patients had an improvement in activity level, although exercise capacity and health status show clinical improvement in 3 of these 5 patients. The feasibility of personalized PA prescription intervention [81] was tested on 2 users. Of these, 1 subject showed excellent adherence until week 10, but the other subject had inconsistent participation. These studies do not demonstrate the effectiveness of the interventions due to their low sample sizes. However, they provide directions toward potential feasible interventions for increasing PA.

Other studies such as the studies by Rabbi et al, Ammann et al, and Spark et al [45,79,82] show significant improvements in PA for their users. A different evaluation metric is used by Short et al [84], which evaluated habit strength of performing PA. The self-reported habit strength for PA increased, which has been considered as an effective improvement for PA intervention.

In both RCT and other studies, several studies have shown significant improvement in the PA of the participants due to personalized interventions. The study by Cook et al [30] showed a significant intervention effect with an increase in active commute and leisure time PA as well as PA in schools for the adolescents. The MyBehavior app evaluation study [45] also stated an increase in walking minutes and calories burnt in nonwalking exercises as compared with the baseline. A study for older adults [83] reported a positive impact on PA, with improvements in endurance, strengthening, stretching, and balance improvements. Similarly, I-Move [38] achieved a small but significant improvement in weekly minutes of MVPA. The study by Partridge et al [68] also reported a statistically significant increase in mean MET-minutes per week. In addition, the total walking days increased in the intervention group as compared with the control group. An increase in weekly minutes of MVPA was reported by Larsen et al [88]. They also reported an increase in the diversity of activities undertaken by participants as compared with the baseline.

A total of 2 studies have reported an improvement in self-reported values but have not observed the same for the objectively measured PA values [35,64]. Several of the studies do observe improvements in PA in the intervention groups; however, these are not statistically significant [9,41,47,74]. Furthermore, 1 study [71] tried to analyze the matched delivery preference and reported no intervention effect with a delivery method (video-only, text-only, or combined) of choice. It also reports that the video-only intervention did not see any improvements.

**Table 3:** Results of individual studies—randomized controlled trials.

Serial #	Paper reference	Dataset size	Variables evaluated	Results
1	Soetens et al [65]	803	Effect of time over increase in PA^a^	PA increases in all groups, time has no significant effect on all completers though has significant effect on those who had low baseline scores for total PA minutes (*P*<.001)
5	Storm et al [7]	790	Strength of habit for PA measured with abbreviated version of Self-Reported Habit Index, self-efficacy, and planning	Self-efficacy (*P*=.1), planning (*P*=.2), and habit strength (*P*=.006) improved in the intervention group
6	Schulz et al [47]	5055	Minutes of PA per day in control, sequential intervention module delivery, and simultaneous module delivery	No statistical difference in sequential and simultaneous delivery for PA or with respect to control group. Sequential delivery could be more effective than simultaneous module delivery after 12 months (*P*=.7)
13	Yom-Tov et al [9]	27	PA minutes per week, change in activity with message policy, change from initial to RL^b^-based learned policy	No statistical difference in treatment and control arm (*P*=.30) for PA minutes per week. Difference in change of activity between initial and learned message policy statistically significant (*P*=.004)
15	Cook et al [30]	555	PA (minutes per week) behavior difference at baseline and postmeasurement for 3 parameters: commuting, leisure time PA, and PA in school	Improvement found in leisure time MVPA^c^ (*P*<.05), for increase in commute by bicycle (around 30 min) (*P*<.01) and total MVPA (*P*<.05)
17	Short et al [84]	724	Minutes per week of MVPA and resistance training score for all 3 arms–3 module interventions delivered monthly, weekly, or single-module	Significant improvement of MVPA across all groups (*P*<.05). Significant improvement in resistance score from monthly 3-module intervention to single module (*P*=.01)
21	Irvine et al [83]	368	Cardiovascular exercises, stretching exercises, strength exercises, balance exercises (all measured in minutes per week), and number of activities	Improvement in intervention group as compared with control in all (*P*<.001)
22	Friederichs et al [38]	4302	Minutes of MVPA per week and number of days ≥30 min activity in I-Move intervention, Active Plus intervention, and control group	I-Move had small but more significant effect than Active Plus in minutes of MVPA per week (*P*=.03 and *P*=.07). I-Move had medium sized effect and Active Plus had large size effect for number of days ≥30 min
23	Blake et al [108]	296	Active travel, moderate activity at work and recreation and vigorous activity at work and recreation in 2 arms for different delivery modes, both with tailored content, one with SMS^d^ and another with email	No significant difference between email and SMS, but significant difference in moderate activity at work (hours per day), with email more effective than SMS (*P*=.24).
25	Hargreaves et al [93]	97	Step count	No difference at baseline and 12 weeks. Significant increase in step count of intervention group between week 12 and week 24 (*P*=.055) but not so significant in comparison group (*P*=.15)
30	Mistry et al [90]	337	PA between the 3 groups–standard care, generic message, and intervention group after 4 weeks	No significant difference between groups for change in PA (*P*>.05)
31	Peels et al [27]	1729	Number of MET^e^ hours in 4 kinds of tailoring: printed, and Web-based (basic and environment-based in each) and control group	Printed (both basic and environmental) had statistically significant increase in MET hours (*P*=.025 and *P*=.31, respectively). No significant increase in both Web-based interventions (*P*=.59 and *P*=.887, respectively)
34	Pyky et al [41]	496	Self-rated health and fitness and leisure time PA	Changes in self-rated fitness and leisure time PA are associated with improved self-rated health (*P*<.026 and *P*<.04, respectively). No significant difference between intervention and control for self-reported daily sitting (*P*=.32) and light housework (but no other leisure time) PA (*P*=.43)
37	Marsaux et al [35]	1607	Objective PA in control group, group with personalized advice on diet and PA (L1 group), L1+phenotype (L2 group) and L2+genotype (L3 group)	No significant difference between control and any of the 3 groups in objective PA level measured (*P*=.73)
38	Alley et al [43]	154	PA (min per week) for 3 groups: control, tailoring only, and tailoring+video coaching group	Significant difference in PA between tailoring+video coaching versus control group (*P*=.01) but no significant difference in PA between the 2 intervention groups (*P*=.54)
39	Mitchell et al [60]	171	Sedentary time, LPA^f^, and MVPA for intervention group with personalized step goals versus control group with generic advice	Decrease in sedentary time, Improvement in LPA and MVPA for both groups (*P*<.005).
41	De Cocker et al [64]	312	Sitting time in 3 groups: control, generic intervention, and tailored intervention	Self-reported total sitting time decreased more in tailored group compared with both generic group (*P*=.002) and control group (*P*=.002). But no significant difference in objectively measured data
47	Kattelmann et al [72]	1639	Total MET-minutes per week estimated from self-reported data	No difference between control and intervention for total MET-minutes per week (*P*=.90). Significant time effect for moderate MET-minutes per week (*P*=.002) and significant time × group × gender effect for vigorous MET-min per week (*P*=.05)
48	Partridge et al [68]	214	Self-reported PA data analyzed as MET-minutes per week	Significant effect of intervention on average MET minutes per week at 12 weeks (*P*=.05). Total PA days (*P*=.003) and number of walking days (*P*=.02) increased in intervention group
49	Walthouwer et al [71]	1419	PA duration in text-tailored, video-tailored, and control arm. In the tailoring group, 2 groups were compared, 1 where preference of user to video/text was matched and another without the matching	No significant difference in condition match/mismatch for PA (*P*=.33). Also, no significant difference for video-tailoring × intervention used (*P*=.83) and text-tailoring × intervention used (*P*=.81)

^a^PA: physical activity.

^b^RL: reinforcement learning

^c^MVPA: moderately vigorous physical activity.

^d^SMS: short messaging service.

^e^MET: metabolic equivalent.

^f^LPA: light physical activity.

**Table 4:** Results of individual studies—nonrandomized controlled trials.

Serial #	Paper reference	Method of study design	Dataset size	Variables evaluated	Result
7	Hermens et al [54]	Single-case experimental study	8	Objectively measured activity behavior (activity level)	5 patients had increased PA^a^ level
11	Rabbi et al [45]	Single case experiment with multiple baseline	16	Minutes of walking per day and calories burnt in nonwalking exercise per day	Intervention had significant effect for walking (*P*<.005) and exercise (*P*<.05)
16	Larsen et al [88]	Observational study	21	Change in minutes of MVPA^b^ using a semistructured interview among adolescent girls after 12 weeks	Statistically significant increase in weekly minutes of MVPA (*P*<.001). Also reported activity types had larger variation than baseline
24	Coolbaugh et al [81]	Feasibility study	2	12 weeks of personalized intervention	Feasibility could not be ascertained
33	Ammann et al [79]	Observational study	803	Weekly total PA minutes across young, middle age, and old age groups	Significant increase in MVPA from baseline for older adults (*P*<.5). All age groups increased weekly PA significantly (*P*<.05) and walking minutes (*P*<.01) over time in intention-to-treat analysis
44	Hales et al [74]	Pilot study and iterative usability study	9	Calories spent during intentional activity for users as compared with baseline	Calories expended increased from baseline but not statistically significant (*P*=.57)
46	Spark et al [82]	Single group, pre- and post-test study	29	Duration of MVPA for participants in initial intervention (6 months), followed by extended contact information (6-12 months) and no contact follow up (12-18 months)	Significant improvement in minutes/day MVPA to 6 months from baseline (*P*=.006) and to 18 months from baseline (*P*=.003)

^a^PA: physical activity.

^b^MVPA: moderately vigorous physical activity.

## Discussion

### Principal Findings

This study provides a review of studies on personalized technology–based interventions for increasing PA. This review adds to the PA literature in several ways. It provides an overview of personalization provided to users in the context of apps that aim at increasing PA. It examines various attributes, which can be personalized for encouraging the user, and identifies the theoretical frameworks used in these studies. This review included all research designs and, thus, provides a comprehensive view of ideas for effectively encouraging PA by means of personalization. We now discuss the review implications with respect to interventions, personalization, user models, theory and guidelines, and results.

### Interventions

The widespread adoption of activity monitoring devices, increasing accuracy of data-driven prediction techniques, and ease of automation all facilitate the use of automated interventions. However, PA changes in patients who are under clinical observation may need to be assessed by a health care expert, leading to manual interventions.

Semiautomated systems combine and thereby aim to provide the best of both worlds—automated and manual interventions. Though these are often specialized for patients [75,80], they can also be available for the general user [2,35]. Having a health care expert–based intervention is less scalable but often necessary for patients under specific medical treatments. An interesting case of semiautomation is seen in the study by Dobrican and Zampunieris [75], where the targets were cardiac patients and the aim was rehabilitation. The doctor was involved for medical advice, but adaptive goals were set based on the European Society of Cardiology guidelines [109]. Note that there are arguments suggested against completely automated systems, for example, they have not been effective in weight loss [2].

Commercial fitness apps designed for the general user could take into account specific requirements of users with clinical conditions, including chronic diseases such as diabetes, who may benefit from such interventions. Current systems would need to include adaptive goal recommendation [54] to offer personalization in light of medical constraints and not just preferences of the users (eg, no swimming for elderly patients). From this review, we observe that user models for patients with chronic diseases are similar. PA guidelines, such as European Society of Cardiology’s guidelines [109] for cardiac patients or by Canadian Diabetes Association [110] and BCT–based design could be incorporated to enable effective behavior change.

### Personalization

Interventions in the included studies were personalized in one or more ways. Recommendations were personalized with respect to goals, activity, or fitness partners. Feedback was personalized with respect to its educational or motivational content and, in some cases, its timing.

Personalization was done either individually or in a category-based manner. The former includes individual models, for example, based on a user’s lifestyle [86], rate of progression [81], and preferences [32] or determined by a health care specialist [80]. In the latter case, category-specific personalization was provided after identifying the most appropriate category for the user. The categories were defined based on BCT [eg, 3] or activity status [eg, 2].

### User Models

User models were created using a variety of different measurements, that is, PA profiles, demographics, medical data, BCT parameters, and contextual information.

Various parameters were used to evaluate PA, and all the profiles aimed to measure 1 or more “dimensions” of PA. An interesting visualization of multidimensional PA was proposed in 1 study [111]. The premise is that PA cannot be judged only on 1 criterion, for example, number of steps or time of vigorous activity, and has multiple dimensions including sedentary time. All the interventions for PA were restricted in their dimensions, and a multidimensional profile would be useful to obtain a holistic view of the user.

User models based on social profiles used the least amount of other contextual parameters. They promoted behavior change through social influence and are promising for both effective persuasion and user modeling. Among the included studies, social profiles were used for buddy matching [74] and also to post status data on social media to promote PA.

Personalized and dynamic user models can be created using the wealth of multimodal user data available from smartphones. Most of the existing apps do not use all the available data. PRO-fit utilized some of the available data sources—the phones’ geolocation, the users’ social network, and the users’ calendar—effectively [56]. By integrating all the available data, a richer profile can be created, and when combined with reinforcement learning techniques, the most effective interventional policies for each user can be learnt. As user behavior may change over time, it is important to employ online learning algorithms that can continuously monitor user models, adapt to their changing lifestyle patterns, and accordingly modify interventions as well.

### Theory and Guidelines

Theory-based studies used BCTs to only make design decisions. Furthermore, 1 study did not completely define all the phases of TTM during the design process but utilized the readiness parameter defined by the model [45]. In addition, incorporation of BCTs was usually done via questionnaires in these studies, which may be infeasible or obtrusive to the user. Thus, automated learning of BCT parameters may be worth exploring. There is preliminary work in this direction. A user’s awareness depends on both the actual and perceived behavior [3]. A study that personalized messages using reinforcement learning concluded that the difference in users’ exercise on a given day could be learnt by the learning algorithm, thus making user behavior predictable [9]. The methodology of utilizing activities of daily life for profiling users and their behavior [86] is another approach for estimating user behavior. User preferences could also be learnt through greedy approaches [86] or through inherent model design [45].

Another problem with methodologies based on BCTs is that they generally set a fixed ideal goal for a user. In contrast, PA guidelines suggest PA progression to prevent fatigue or muscular injuries. The generic goals of 60 min of PA or 10,000 steps may be too difficult and hence demotivating to a user who is sedentary or has clinical complications. Such users often require help, in the form of intermediate goals, to reach the final goal. PA guidelines can be utilized in such cases. There are attempts in studies [87] to use PA guidelines while using BCT for motivating users. Another study [78] also encourages its users, that is, cancer patients, to follow guidelines set by American Cancer Association [110] while planning their PA.

As identified across the PA literature, an “intention-behavior” gap exists among users. This poses the classic problem that although users are motivated and have intentions to increase their PA, they are not sufficiently active. Many studies were based on BCTs. However, healthy lifestyle induced during the intervention does not ensure that the user does not go back to a sedentary lifestyle after the intervention [54]. The sustained effects of interventions were not evaluated by all the studies but only by a few studies (e.g. [45,60,82]). Habit strength and formation has been addressed and evaluated in the study by Storm et al [7]. It is important that the sustained long-term effects of intervention are analyzed, as it would help to identify effective methods of promoting PA.

### Results of Individual Studies

Direct positive results demonstrating the effectiveness of personalized interventions have been observed in a diverse set of studies—there are studies implementing data-driven automated systems [45], which recommend activities, whereas there are also studies which provide only personalized educational and motivational content [30]. These results indicate that activity or definite goal recommendation is not required for an effective personalized PA intervention. Effectively personalized motivational and educational content can help induce behavior change among the participants as well. It is also interesting to note that most of the studies with significant improvements are based on theoretical models (e.g.[7,30,54,88]). However, most of these studies also use self-reported values and collect data through questionnaires (e.g.[79,84,108]).

The self-reported PA values need to be considered with caution. As observed in the studies by Marsaux et al and De Cocker et al [35,64], there can be different results when self-reported and objectively measured data are compared. Thus, positive results obtained by interventions based on self-reported data need to be evaluated with objectively measured data through accelerometers and sensors. However, it can be inferred by the positive results obtained through BCT that their incorporation could help users, even if the users’ perceived PA level is incorrect. This makes it worthwhile to find ways of incorporating BCTs and theoretical guidelines in other data-driven–based interventions. However, it also needs to be noted that even the studies that do not show significant improvements use BCTs [47,74,90].

Studies have evaluated not just the PA metrics but also the intervention delivery mediums. The intervention delivery was found to not matter in the cases of video versus text [71] and SMS versus email [108]. However, a difference was found in the case of print versus Web-based intervention [27]. It is possible that the print medium was found to be effective as the participants were adults over the age of 50 years. However, further studies need to be performed to analyze the differences between intervention delivery mediums and their effects on the users.

The sample sizes of the studies reviewed vary considerably. The studies have also not been analyzed for quality to recommend future directions. However, our review indicates that there is scope for more rigorous evaluation in terms of intervention delivery, personalization, and intervention method. Many studies in the review perform pilot studies or feasibility studies or identify RCT protocols, which are yet to be completely evaluated. Evaluation of various systems to identify the effectiveness of intervention medium (along with the personalization aspect) in motivating users could be useful.

### Limitations

This review was restricted to specific databases and an appropriate search query. It is possible that some studies may have been left out due to their journal or indexing bias. In addition, the search was restricted to a time frame that was considered relevant for the personalization aspect of the study and could again have led to studies being left out of the review. Moreover, as this is a scoping review, we have included studies without quality analysis and also studies without any evaluation. Though it helps identify the breadth of research, as the quality of studies is not assessed, the gaps identified may not be completely accurate.

### Conclusions

This study provides a comprehensive review of personalized technology–based interventions, as recommendations or feedback, for promoting PA. Overall, the studies show that these interventions for increasing PA are more effective when they are personalized, compared with a “one size fits all” generic advice. Gaps have been identified in several aspects, such as in the development of a multidimensional user model and the use of behavioral theory in automated personalization. On the basis of these gaps, research directions for improving the efficacy of personalized technology–based interventions have been suggested.

## Appendix

Multimedia Appendix 1 Overview of studies in the review.

Multimedia Appendix 2 User model parameters for studies in the review.

## Abbreviations

BCT: behavior change technique
CDC: Centers for Disease Control and Prevention
CVD: cardiovascular disease
HAPA: health action process approach
HR: heart rate
LPA: light physical activity
MET: metabolic equivalent
MVPA: moderately vigorous physical activity
PA: physical activity
RCT: randomized controlled trial
RL: reinforcement learning
RNN: recurrent neural networks
SCT: social cognitive theory
SMS: short messaging service
SVM: support vector machine
TPB: Theory of Planned Behavior
TTM: Trans-Theoretical Model

## References

[1] World Health Organization.

[2] Martin CK, Gilmore LA, Apolzan JW, Myers CA, Thomas DM, Redman LM (2016). Smartloss: a personalized mobile health intervention for weight management and health promotion. JMIR Mhealth Uhealth.

[3] Klein MC, Manzoor A, Mollee JS (2017). Active2Gether: A personalized m-health intervention to encourage physical activity. Sensors (Basel).

[4] Forastiere M, De Pietro G, Sannino G (2016). An mHealth Application for a Personalized Monitoring of One’s Own Wellness: Design and Development. Smart Innovation, Systems and Technologies.

[5] Kerner C, Goodyear V (2017). The motivational impact of wearable healthy lifestyle technologies: a self-determination perspective on Fitbits with adolescents. Am J Heal Educ.

[6] Mukhtar H (2016). Using persuasive recommendations in wellness applications based upon user activities. Int J Adv Comput Sci Appl.

[7] Storm V, Dörenkämper J, Reinwand DA, Wienert J, De Vries H, Lippke S (2016). Effectiveness of a web-based computer-tailored multiple-lifestyle intervention for people interested in reducing their cardiovascular risk: a randomized controlled trial. J Med Internet Res.

[8] Kroeze W, Werkman A, Brug J (2006). A systematic review of randomized trials on the effectiveness of computer-tailored education on physical activity and dietary behaviors. Ann Behav Med.

[9] Yom-Tov E, Feraru G, Kozdoba M, Mannor S, Tennenholtz M, Hochberg I (2017). Encouraging physical activity in patients with diabetes: intervention using a reinforcement learning system. J Med Internet Res.

[10] Joseph RP, Durant NH, Benitez TJ, Pekmezi DW (2014). Internet-based physical activity interventions. Am J Lifestyle Med.

[11] Baker PR, Francis DP, Soares J, Weightman AL, Foster C (2015). Community wide interventions for increasing physical activity. Cochrane Database Syst Rev.

[12] Rogers MA, Lemmen K, Kramer R, Mann J, Chopra V (2017). Internet-delivered health interventions that work: systematic review of meta-analyses and evaluation of website availability. J Med Internet Res.

[13] Baker P, Dobbins M, Soares J, Francis D, Weightman A, Costello J (2015). Public health interventions for increasing physical activity in children, adolescents and adults: an overview of systematic reviews. Cochrane Database Syst Rev.

[14] Saunders DH, Sanderson M, Brazzelli M, Greig CA, Mead GE (2013). Physical fitness training for stroke patients. Cochrane Database Syst Rev.

[15] Duff OM, Walsh DM, Furlong BA, O'Connor NE, Moran KA, Woods CB (2017). Behavior change techniques in physical activity eHealth interventions for people with cardiovascular disease: systematic review. J Med Internet Res.

[16] Claes J, Buys R, Woods C, Briggs A, Geue C, Aitken M, Moyna N, Moran K, McCaffrey N, Chouvarda I, Walsh D, Budts W, Filos D, Triantafyllidis A, Maglaveras N, Cornelissen VA (2017). PATHway I: design and rationale for the investigation of the feasibility, clinical effectiveness and cost-effectiveness of a technology-enabled cardiac rehabilitation platform. BMJ Open.

[17] op den Akker H, Jones VM, Hermens HJ (2014). Tailoring real-time physical activity coaching systems: a literature survey and model. User Model User-Adap Inter.

[18] Fan H, Poole MS (2006). What is personalization? Perspectives on the design and implementation of personalization in information systems. J Org Comp Elect Com.

[19] Hawkins RP, Kreuter M, Resnicow K, Fishbein M, Dijkstra A (2008). Understanding tailoring in communicating about health. Health Educ Res.

[20] Arksey H, O'Malley L (2005). Scoping studies: towards a methodological framework. Int J Soc Res Methodol.

[21] Oosterom-Calo R, Te Velde SJ, Stut W, Brug J (2015). Development of Motivate4Change using the intervention mapping protocol: an interactive technology physical activity and medication adherence promotion program for hospitalized heart failure patients. JMIR Res Protoc.

[22] Liu C, Chan C (2016). A fuzzy logic prompting mechanism based on pattern recognition and accumulated activity effective index using a smartphone embedded sensor. Sensors (Basel).

[23] Janols R, Guerrero E, Lindgren H (2017). A pilot study on personalised coaching to increase older adults' physical and social activities. Advances in Intelligent Systems and Computing.

[24] Lindgren H, Guerrero E, Janols R (2017). Personalised Persuasive Coaching to Increase Older Adults’ Physical and Social Activities: A Motivational Model. Advances in Practical Applications of Cyber-Physical Multi-Agent Systems: The PAAMS Collection.

[25] Boekhout JM, Berendsen BA, Peels DA, Bolman CA, Lechner L (2018). Evaluation of a computer-tailored healthy ageing intervention to promote physical activity among single older adults with a chronic disease. Int J Environ Res Public Health.

[26] Peels DA, de Vries H, Bolman C, Golsteijn RH, van Stralen MM, Mudde AN, Lechner L (2013). Differences in the use and appreciation of a web-based or printed computer-tailored physical activity intervention for people aged over 50 years. Health Educ Res.

[27] Peels DA, Hoogenveen RR, Feenstra TL, Golsteijn RH, Bolman C, Mudde AN, Wendel-Vos GC, de Vries H, Lechner L (2014). Long-term health outcomes and cost-effectiveness of a computer-tailored physical activity intervention among people aged over fifty: modelling the results of a randomized controlled trial. BMC Public Health.

[28] Van Stralen MM, Kok G, de Vries H, Mudde AN, Bolman C, Lechner L (2008). The Active plus protocol: systematic development of two theory- and evidence-based tailored physical activity interventions for the over-fifties. BMC Public Health.

[29] Klein MC, Manzoor A, Middelweerd A, Mollee JS, te Velde SJ (2015). Encouraging physical activity via a personalized mobile system. IEEE Internet Comput.

[30] Cook TL, De Bourdeaudhuij I, Maes L, Haerens L, Grammatikaki E, Widhalm K, Kwak L, Plada M, Moreno LA, Zampelas A, Tountas Y, Manios Y (2014). Moderators of the effectiveness of a web-based tailored intervention promoting physical activity in adolescents: the HELENA Activ-O-Meter. J Sch Health.

[31] De Bourdeaudhuij I, Maes L, De Henauw S, De Vriendt T, Moreno LA, Kersting M, Sarri K, Manios Y, Widhalm K, Sjöstrom M, Ruiz JR, Haerens L, HELENA Study Group (2010). Evaluation of a computer-tailored physical activity intervention in adolescents in six European countries: the Activ-O-Meter in the HELENA intervention study. J Adolesc Health.

[32] Fahim M, Idris M, Ali R, Nugent C, Kang B, Huh E, Lee S (2014). ATHENA: a personalized platform to promote an active lifestyle and wellbeing based on physical, mental and social health primitives. Sensors (Basel).

[33] Ali R, Siddiqi M, Kang B, Lee S (2015). KARE: A Hybrid Reasoning Approach for Promoting Active Lifestyle. Proceedings of the 9th International Conference on Ubiquitous Information Management and Communication.

[34] Marsaux CF, Celis-Morales C, Fallaize R, Macready AL, Kolossa S, Woolhead C, O'Donovan CB, Forster H, Navas-Carretero S, San-Cristobal R, Lambrinou C, Moschonis G, Surwillo A, Godlewska M, Goris A, Hoonhout J, Drevon CA, Manios Y, Traczyk I, Walsh MC, Gibney ER, Brennan L, Martinez JA, Lovegrove JA, Gibney MJ, Daniel H, Mathers JC, Saris WH (2015). Effects of a web-based personalized intervention on physical activity in European adults: a randomized controlled trial. J Med Internet Res.

[35] Marsaux CF, Celis-Morales C, Livingstone KM, Fallaize R, Kolossa S, Hallmann J, San-Cristobal R, Navas-Carretero S, O'Donovan CB, Woolhead C, Forster H, Moschonis G, Lambrinou C, Surwillo A, Godlewska M, Hoonhout J, Goris A, Macready AL, Walsh MC, Gibney ER, Brennan L, Manios Y, Traczyk I, Drevon CA, Lovegrove JA, Martinez JA, Daniel H, Gibney MJ, Mathers JC, Saris WH (2016). Changes in physical activity following a genetic-based internet-delivered personalized intervention: randomized controlled trial (Food4Me). J Med Internet Res.

[36] Celis-Morales C, Livingstone KM, Marsaux CF, Forster H, O'Donovan CB, Woolhead C, Macready AL, Fallaize R, Navas-Carretero S, San-Cristobal R, Kolossa S, Hartwig K, Tsirigoti L, Lambrinou CP, Moschonis G, Godlewska M, SurwiÅ‚Å‚o A, Grimaldi K, Bouwman J, Daly EJ, Akujobi V, O'Riordan R, Hoonhout J, Claassen A, Hoeller U, Gundersen TE, Kaland SE, Matthews JN, Manios Y, Traczyk I, Drevon CA, Gibney ER, Brennan L, Walsh MC, Lovegrove JA, Alfredo MJ, Saris WH, Daniel H, Gibney M, Mathers JC (2015). Design and baseline characteristics of the Food4Me study: a web-based randomised controlled trial of personalised nutrition in seven European countries. Genes Nutr.

[37] Friederichs SA, Oenema A, Bolman C, Guyaux J, Van Keulen HM, Lechner L (2014). I Move: systematic development of a web-based computer tailored physical activity intervention, based on motivational interviewing and self-determination theory. BMC Public Health.

[38] Friederichs SA, Oenema A, Bolman C, Lechner L (2016). Motivational interviewing and self-determination theory in a web-based computer tailored physical activity intervention: a randomized controlled trial. Psychol Health.

[39] Ahola R, Pyky R, Jämsä T, Mäntysaari M, Koskimäki H, Ikäheimo TM, Huotari M, Röning J, Heikkinen HI, Korpelainen R (2013). Gamified physical activation of young men--a Multidisciplinary Population-Based Randomized Controlled Trial (MOPO study). BMC Public Health.

[40] Jauho A, Pyky R, Ahola R, Kangas M, Virtanen P, Korpelainen R, Jämsä T (2015). Effect of wrist-worn activity monitor feedback on physical activity behavior: a randomized controlled trial in Finnish young men. Prev Med Rep.

[41] Pyky R, Koivumaa-Honkanen H, Leinonen A, Ahola R, Hirvonen N, Enwald H, Luoto T, Ferreira E, Ikäheimo TM, Keinänen-Kiukaanniemi S, Mäntysaari M, Jämsä T, Korpelainen R (2017). Effect of tailored, gamified, mobile physical activity intervention on life satisfaction and self-rated health in young adolescent men: a population-based, randomized controlled trial (MOPO study). Comput Human Behav.

[42] Alley S, Jennings C, Plotnikoff RC, Vandelanotte C (2014). My Activity Coach - using video-coaching to assist a web-based computer-tailored physical activity intervention: a randomised controlled trial protocol. BMC Public Health.

[43] Alley S, Jennings C, Plotnikoff RC, Vandelanotte C (2016). Web-based video-coaching to assist an automated computer-tailored physical activity intervention for inactive adults: a randomized controlled trial. J Med Internet Res.

[44] Rabbi M, Pfammatter A, Zhang M, Spring B, Choudhury T (2015). Automated personalized feedback for physical activity and dietary behavior change with mobile phones: a randomized controlled trial on adults. JMIR Mhealth Uhealth.

[45] Rabbi M, Aung MH, Zhang M, Choudhury T (2015). MyBehavior: automatic personalized health feedback from user behaviors and preferences using smartphones. Proceedings of the 2015 ACM International Joint Conference on Pervasive and Ubiquitous Computing.

[46] Schulz DN, Kremers SP, van Osch LA, Schneider F, van Adrichem MJ, de Vries VH (2011). Testing a Dutch web-based tailored lifestyle programme among adults: a study protocol. BMC Public Health.

[47] Schulz DN, Kremers SP, Vandelanotte C, van Adrichem MJ, Schneider F, Candel MJ, de Vries H (2014). Effects of a web-based tailored multiple-lifestyle intervention for adults: a two-year randomized controlled trial comparing sequential and simultaneous delivery modes. J Med Internet Res.

[48] Williams QI, Gunn AH, Beaulieu JE, Benas BC, Buley B, Callahan LF, Cantrell J, Genova AP, Golightly YM, Goode AP, Gridley CI, Gross MT, Heiderscheit BC, Hill CH, Huffman KM, Kline A, Schwartz TA, Allen KD (2015). Physical therapy vs. internet-based exercise training (PATH-IN) for patients with knee osteoarthritis: study protocol of a randomized controlled trial. BMC Musculoskelet Disord.

[49] Brooks MA, Beaulieu JE, Severson HH, Wille CM, Cooper D, Gau JM, Heiderscheit BC (2014). Web-based therapeutic exercise resource center as a treatment for knee osteoarthritis: a prospective cohort pilot study. BMC Musculoskelet Disord.

[50] Chatzitofis A, Zarpalas D, Filos D, Triantafyllidis A, Chouvarda I, Maglaveras N (2017). Technological Module for Unsupervised, Personalized Cardiac Rehabilitation Exercising.

[51] Claes J, Buys R, Woods C, Briggs A, Geue C, Aitken M, Moyna N, Moran K, McCaffrey N, Chouvarda I, Walsh D, Budts W, Filos D, Triantafyllidis A, Maglaveras N, Cornelissen VA (2017). PATHway I: design and rationale for the investigation of the feasibility, clinical effectiveness and cost-effectiveness of a technology-enabled cardiac rehabilitation platform. BMJ Open.

[52] Triantafyllidis A, Filos D, Buys R, Claes J, Cornelissen V, Kouidi E, Chatzitofis A, Zarpalas D, Daras P, Walsh D, Woods C, Moran K, Maglaveras N, Chouvarda I (2018). Computerized decision support for beneficial home-based exercise rehabilitation in patients with cardiovascular disease. Comput Methods Programs Biomed.

[53] Cabrita M, Akker HD, Achterkamp R, Hermens H, Vollenbroek-Hutten M (2014). Automated Personalized Goal-setting in an Activity Coaching Application. Proceedings of the 3rd International Conference on Sensor Networks - Volume 1: SENSORNETS.

[54] Hermens H, Op den Akker H, Tabak M, Wijsman J, Vollenbroek M (2014). Personalized Coaching Systems to support healthy behavior in people with chronic conditions. J Electromyogr Kinesiol.

[55] Op den Akker H, Cabrita M, Op den Akker R, Jones VM, Hermens HJ (2015). Tailored motivational message generation: a model and practical framework for real-time physical activity coaching. J Biomed Inform.

[56] Dharia S, Jain V, Patel J, Vora J, Yamauchi R, Eirinaki M (2016). PRO-Fitxercise with friends. Proceedings of the 2016 IEEE/ACM International Conference on Advances in Social Networks Analysis and Mining.

[57] Dharia S, Eirinaki M, Jain V, Patel J, Varlamis I, Vora J, Yamauchi R (2017). Social recommendations for personalized fitness assistance. Pers Ubiquit Comput.

[58] Dharia S, Jain V, Patel J, Vora J, Chawla S, Eirinaki M (2016). PRO-Fit: A personalized fitness assistant framework.

[59] Mitchell BL, Lewis NR, Smith AE, Rowlands AV, Parfitt G, Dollman J (2014). Rural Environments and Community Health (REACH): a randomised controlled trial protocol for an online walking intervention in rural adults. BMC Public Health.

[60] Mitchell BL, Smith AE, Rowlands AV, Fraysse F, Parfitt G, Lewis NR, Dollman J (2018). Promoting physical activity in rural Australian adults using an online intervention. J Sci Med Sport.

[61] Reinwand D, Kuhlmann T, Wienert J, de Vries H, Lippke S (2013). Designing a theory- and evidence-based tailored eHealth rehabilitation aftercare program in Germany and the Netherlands: study protocol. BMC Public Health.

[62] Martin CK, Miller AC, Thomas DM, Champagne CM, Han H, Church T (2015). Efficacy of SmartLoss, a smartphone-based weight loss intervention: results from a randomized controlled trial. Obesity (Silver Spring).

[63] De Cocker K, De Bourdeaudhuij I, Cardon G, Vandelanotte C (2015). Theory-driven, web-based, computer-tailored advice to reduce and interrupt sitting at work: development, feasibility and acceptability testing among employees. BMC Public Health.

[64] De Cocker K, De Bourdeaudhuij I, Cardon G, Vandelanotte C (2016). The effectiveness of a web-based computer-tailored intervention on workplace sitting: a randomized controlled trial. J Med Internet Res.

[65] Soetens KC, Vandelanotte C, de Vries VH, Mummery KW (2014). Using online computer tailoring to promote physical activity: a randomized trial of text, video, and combined intervention delivery modes. J Health Commun.

[66] Vandelanotte C, Short C, Plotnikoff RC, Hooker C, Canoy D, Rebar A, Alley S, Schoeppe S, Mummery WK, Duncan MJ (2015). TaylorActive--Examining the effectiveness of web-based personally-tailored videos to increase physical activity: a randomised controlled trial protocol. BMC Public Health.

[67] Hebden L, Balestracci K, McGeechan K, Denney-Wilson E, Harris M, Bauman A, Allman-Farinelli M (2013). 'TXT2BFiT' a mobile phone-based healthy lifestyle program for preventing unhealthy weight gain in young adults: study protocol for a randomized controlled trial. Trials.

[68] Partridge SR, McGeechan K, Hebden L, Balestracci K, Wong AT, Denney-Wilson E, Harris MF, Phongsavan P, Bauman A, Allman-Farinelli M (2015). Effectiveness of a mHealth lifestyle program with telephone support (TXT2BFiT) to prevent unhealthy weight gain in young adults: randomized controlled trial. JMIR Mhealth Uhealth.

[69] Walthouwer MJ, Oenema A, Lechner L, de Vries H (2015). Use and effectiveness of a video- and text-driven web-based computer-tailored intervention: randomized controlled trial. J Med Internet Res.

[70] Walthouwer MJ, Oenema A, Soetens K, Lechner L, de Vries H (2017). Implementation of web-based interventions by Dutch occupational health centers. Health Promot Int.

[71] Walthouwer MJ, Oenema A, Soetens K, Lechner L, De Vries H (2013). Systematic development of a text-driven and a video-driven web-based computer-tailored obesity prevention intervention. BMC Public Health.

[72] Kattelmann KK, White AA, Greene GW, Byrd-Bredbenner C, Hoerr SL, Horacek TM, Kidd T, Colby S, Phillips BW, Koenings MM, Brown ON, Olfert M, Shelnutt KP, Morrell JS (2014). Development of Young Adults Eating and Active for Health (YEAH) internet-based intervention via a community-based participatory research model. J Nutr Educ Behav.

[73] Kattelmann KK, Bredbenner CB, White AA, Greene GW, Hoerr SL, Kidd T, Colby S, Horacek TM, Phillips BW, Koenings MM, Brown ON, Olfert MD, Shelnutt KP, Morrell JS (2014). The effects of Young Adults Eating and Active for Health (YEAH): a theory-based Web-delivered intervention. J Nutr Educ Behav.

[74] Hales S, Turner-McGrievy G, Fahim A, Freix A, Wilcox S, Davis RE, Huhns M, Valafar H (2016). A mixed-methods approach to the development, refinement, and pilot testing of social networks for improving healthy behaviors. JMIR Hum Factors.

[75] Dobrican R, Zampunieris D (2016). A Proactive Solution, using Wearable and Mobile Applications, for Closing the Gap between the Rehabilitation Team and Cardiac Patients.

[76] Tseng J, Lin BH, Lin YF, Tseng V, Day ML, Wang SH, Lo KR, Yang YC (2015). An interactive healthcare system with personalized diet and exercise guideline recommendation.

[77] Ahire S, Khanuja H (2015). A Personalized Framework for Health Care Recommendation.

[78] Lee MK, Park H, Yun YH, Chang YJ (2013). Development and formative evaluation of a web-based self-management exercise and diet intervention program with tailored motivation and action planning for cancer survivors. JMIR Res Protoc.

[79] Ammann R, Vandelanotte C, de Vries H, Mummery WK (2013). Can a website-delivered computer-tailored physical activity intervention be acceptable, usable, and effective for older people?. Health Educ Behav.

[80] Codreanu I, Florea A (2015). A Proposed Serious Game Architecture to Self-Management HealthCare for Older Adults.

[81] Coolbaugh CL, Raymond SC, Hawkins DA (2015). Feasibility of a dynamic web guidance approach for personalized physical activity prescription based on daily information from wearable technology. JMIR Res Protoc.

[82] Spark LC, Fjeldsoe BS, Eakin EG, Reeves MM (2015). Efficacy of a text message-delivered extended contact intervention on maintenance of weight loss, physical activity, and dietary behavior change. JMIR Mhealth Uhealth.

[83] Irvine AB, Gelatt VA, Seeley JR, Macfarlane P, Gau JM (2013). Web-based intervention to promote physical activity by sedentary older adults: randomized controlled trial. J Med Internet Res.

[84] Short CE, Rebar A, James EL, Duncan MJ, Courneya KS, Plotnikoff RC, Crutzen R, Vandelanotte C (2017). How do different delivery schedules of tailored web-based physical activity advice for breast cancer survivors influence intervention use and efficacy?. J Cancer Surviv.

[85] Ali R, Afzal M, Hussain M, Ali M, Siddiqi MH, Lee S, Ho KB (2016). Multimodal hybrid reasoning methodology for personalized wellbeing services. Comput Biol Med.

[86] Lim C, Kim ZM, Choi H (2017). Developing a mobile wellness management system for healthy lifestyle by analyzing daily living activities. Stud Health Technol Inform.

[87] Moreau M, Gagnon M, Boudreau F (2015). Development of a fully automated, web-based, tailored intervention promoting regular physical activity among insufficiently active adults with type 2 diabetes: integrating the I-change model, self-determination theory, and motivational interviewing components. JMIR Res Protoc.

[88] Larsen B, Benitez T, Cano M, Dunsiger SS, Marcus BH, Mendoza-Vasconez A, Sallis JF, Zive M (2018). Web-based physical activity intervention for Latina adolescents: feasibility, acceptability, and potential efficacy of the Niñas Saludables study. J Med Internet Res.

[89] Rajanna V, Behera D, Goldberg D, Hammond T (2014). Step Up Life: A Context Aware Health Assistant. Proceedings of the Third ACM SIGSPATIAL International Workshop on the Use of GIS in Public Health.

[90] Mistry CD, Sweet SN, Rhodes RE, Latimer-Cheung AE (2015). Text2Plan: exploring changes in the quantity and quality of action plans and physical activity in a text messaging intervention. Psychol Health.

[91] Centers for Disease Control and Prevention.

[92] Haskell WL, Lee I, Pate RR, Powell KE, Blair SN, Franklin BA, Macera CA, Heath GW, Thompson PD, Bauman A (2007). Physical activity and public health: updated recommendation for adults from the American College of Sports Medicine and the American Heart Association. Med Sci Sports Exerc.

[93] Hargreaves EA, Mutrie N, Fleming JD (2016). A web-based intervention to encourage walking (StepWise): pilot randomized controlled trial. JMIR Res Protoc.

[94] Twardowski B, Ryzko D (2015). IoT and Context-Aware Mobile Recommendations Using Multi-agent Systems.

[95] Varadharajan V, Kannav V, Pasala A (2016). Sensor based coaching for a fit and healthy society.

[96] Harris JA, Benedict FG (1918). A biometric study of human basal metabolism. Proc Natl Acad Sci U S A.

[97] Boudreau F, Walthouwer MJL, De Vries H, Dagenais GR, Turbide G, Bourlaud A, Moreau M, Côté J, Poirier P (2015). Rationale, design and baseline characteristics of a randomized controlled trial of a web-based computer-tailored physical activity intervention for adults from Quebec City. BMC Public Health.

[98] Kwasnicka D, Vandelanotte C, Rebar A, Gardner B, Short C, Duncan M, Crook D, Hagger MS (2017). Comparing motivational, self-regulatory and habitual processes in a computer-tailored physical activity intervention in hospital employees - protocol for the PATHS randomised controlled trial. BMC Public Health.

[99] Fogg BJ (2009). A behavior model for persuasive design. Proceedings of the 4th International Conference on Persuasive Technology.

[100] Bandura A (1991). Social cognitive theory of self-regulation. Organ Behav Hum Decis Process.

[101] Prochaska JO, Gellman MD, Turner JR (2013). Transtheoretical Model of Behavior Change. Encyclopedia of Behavioral Medicine.

[102] Ajzen I (1991). The theory of planned behavior. Organ Behav Hum Decis Process.

[103] de Vries H, Mudde A, Leijs I, Charlton A, Vartiainen E, Buijs G, Clemente MP, Storm H, González NA, Nebot M, Prins T, Kremers S (2003). The European Smoking Prevention Framework Approach (EFSA): an example of integral prevention. Health Educ Res.

[104] Mitchie S, Atkins L, West R (2014). The Behaviour Change Wheel: A Guide To Designing Interventions.

[105] Rosenstock IM (1988). Adoption and maintenance of lifestyle modifications. Am J Prev Med.

[106] Rollnick S, Miller W, Butler C, Aloia M (2009). Motivational interviewing in health care: helping patients change behavior. J Chronic Obstr Pulm Dis.

[107] Schwarzer R, Lippke S, Luszczynska A (2011). Mechanisms of health behavior change in persons with chronic illness or disability: the Health Action Process Approach (HAPA). Rehabil Psychol.

[108] Blake H, Suggs LS, Coman E, Aguirre L, Batt ME (2017). Active8! Technology-based intervention to promote physical activity in hospital employees. Am J Health Promot.

[109] McMurray JJ, Adamopoulos S, Anker SD, Auricchio A, Böhm M, Dickstein K, Falk V, Filippatos G, Fonseca C, Gomez-Sanchez MA, Jaarsma T, Køber L, Lip GY, Maggioni AP, Parkhomenko A, Pieske BM, Popescu BA, Rønnevik PK, Rutten FH, Schwitter J, Seferovic P, Stepinska J, Trindade PT, Voors AA, Zannad F, Zeiher A, Bax JJ, Baumgartner H, Ceconi C, Dean V, Deaton C, Fagard R, Funck-Brentano C, Hasdai D, Hoes A, Kirchhof P, Knuuti J, Kolh P, McDonagh T, Moulin C, Popescu BA, Reiner Z, Sechtem U, Sirnes PA, Tendera M, Torbicki A, Vahanian A, Windecker S, McDonagh T, Sechtem U, Bonet LA, Avraamides P, Ben LH, Brignole M, Coca A, Cowburn P, Dargie H, Elliott P, Flachskampf FA, Guida GF, Hardman S, Iung B, Merkely B, Mueller C, Nanas JN, Nielsen OW, Orn S, Parissis JT, Ponikowski P, Task Force for the Diagnosis and Treatment of Acute and Chronic Heart Failure 2012 of the European Society of Cardiology (2012). ESC guidelines for the diagnosis and treatment of acute and chronic heart failure 2012: The Task Force for the Diagnosis and Treatment of Acute and Chronic Heart Failure 2012 of the European Society of Cardiology. Developed in collaboration with the Heart Failure Association (HFA) of the ESC. Eur J Heart Fail.

[110] Booth G, Cheng AYY, Canadian Diabetes Association Clinical Practice Guidelines Expert Committee (2013). Canadian Diabetes Association 2013 clinical practice guidelines for the prevention and management of diabetes in Canada. Methods. Can J Diabetes.

[111] Peacock OJ, Western MJ, Batterham AM, Stathi A, Standage M, Tapp A, Bennett P, Thompson D (2015). Multidimensional individualised Physical ACTivity (Mi-PACT)--a technology-enabled intervention to promote physical activity in primary care: study protocol for a randomised controlled trial. Trials.

