# Supplementary material for: User Models for Personalized Physical Activity Interventions: Scoping Review
Source: JMIR Mhealth Uhealth. 2019 Jan 16;7(1):e11098. doi: 10.2196/11098 (PMC6352015; doi:10.2196/11098)
Supplement: Multimedia Appendix 1 [file mhealth_v7i1e11098_app1.pdf]

This is a Multimedia Appendix to a full manuscript published in the J Med Internet Res. For full copyright and citation information see http://dx.doi.org/10.2196/11098.

Table: Overview of studies

Serial number	Paper reference	Objective	Intervention presentation	Intervention generation	Intervention generation technique	Theoretical model	Personalization	User model
1	Vandelanotte et al [1]	Healthy lifestyle	Website	Automatic	Knowledge-based	TPB[a] and TTM[b]	Goal and activity recommendation and educational and motivational content	PA[c] profile, demographics, BCT[d] parameters, and contextual information
2	Ahire et al [2]	Healthy lifestyle : diet and exercise	Website	Automatic	Knowledge-based and data-driven–decision tree	—	Activity recommendation	PA profile, demographics, medical data, and contextual information
3	Mukhtar [3]	Healthy lifestyle:	Mobile app	Automatic	Knowledge-based	Fogg’s Behav	Activity recomme	PA profile,

		exercise and diet	widget			ior Model and various clinical guidelines for health care	ndation and intervention timing	demographics, medical data, and contextual information
4	Tseng et al [4]	Healthy lifestyle : exercise and diet	Website and mobile app	Semiautomated	Knowledge-based	—	Goal and activity recommendation	PA profile and medical data
5	Storm et al [5]	Healthy lifestyle : exercise and fruits and vegetable consumption	Website	Automatic	Knowledge-based	HAPA[e]	Educational and motivational content	PA profile, demographics, and BCT parameters
6	Schulz et al [6]	Healthy lifestyle : PA, diet, alcohol, and smoking	Website	Automatic	Knowledge-based	I-Change Model	Motivational content	PA profile, demographics, medical data,

								and contextual information
7	Hermens et al [7]	Healthy lifestyle : personalized coaching system	Mobile app	Automatic	Combined knowledge-based and data-driven	Stage of change model	Goal and activity recommendation, motivational content, intervention timing	PA profile, demographics, BCT parameters, and contextual information
8	Lee et al [8]	Healthy lifestyle : exercise and diet	Website	Automatic	Knowledge-based	American Cancer Society guidelines and TTM	Goal and activity recommendation and motivational and educational content	PA profile, demographics, BCT parameters, and contextual information
9	Fahim et al [9]	Increase PA	Any device from API[f]	Automatic	Combined knowledge-based and data-driven	Centers for Disease Contro	Activity recommendation	PA profile, demographics, and

						l and Prevention guidelines		contextual information
10	Dharia et al [10]	Increase PA	Mobile app	Automatic	Data-driven:collaborative filtering	—	Activity and fitness partner recommendation and intervention timing	PA profile, demographics, and contextual information
11	Rabbi et al [11]	Increase PA	Mobile app	Automatic	Data-driven:multi armed bandit and pareto frontier	Principle of SCT[g] and Fogg's Behavior Model and TTM	Goal and activity recommendation	PA profile, demographics, and contextual information
12	Twardowski et al [12]	Increase PA	[h]	Automatic	Data-driven:recommendation system	—	Goal and activity recommendation	PA profile, demographics, medical data, and context

								ual information
13	Yom-Tov et al [13]	Increase PA	SMS[i]	Automatic	Data-driven reinforcement learning	—	Motivational content	PA profile and demographics
14	Lim et al [14]	Increase PA	Mobile app	Automatic	Data-driven-RNN[j]	—	Activity recommendation and intervention timing	PA profile and contextual information
15	Cook et al [15]	Increase PA	Website	Automatic	Knowledge-based	TPB and SCT	Educational and motivational content	PA profile, demographics, and BCT parameters
16	Larsen et al [16]	Increase PA	Website	Automatic	Knowledge-based	SCT and TTM	Educational content	PA profile, demographics, medical data, and BCT

								parameters
17	Short et al [17]	Increase PA	Print, website	Automatic	Knowledge-based	SCT	Educational and motivational content	PA profile, demographics, medical data, BCT parameters, and contextual information
18	Boudreau et al [18]	Increase PA	Website	Automatic	Knowledge-based	I-Change model	Educational and motivational content	PA profile, demographics, and BCT parameters
19	Moreau et al [19]	Increase PA	Website	Automatic	Knowledge-based	SCT, TPB, and PA guidelines by Canadian	Educational and motivational content	PA profile, demographics, and BCT parameters

						Diabetes Association		
20	Rajanna et al [20]	Increase PA	Mobile app	Automatic	Knowledge-based	Fogg's Behavior Model	Goal and activity recommendation and intervention timing	PA profile, demographics, and contextual information
21	Irvine et al [21]	Increase PA	Website and printout	Automatic	Knowledge-based	Stage of Change Model	Activity recommendation and educational content	PA profile and BCT parameters
22	Friederichs et al [22]	Increase PA	Website	Automatic	Knowledge-based	SDT[k] and motivational interviewing	Educational and motivational content	PA profile, demographics, and BCT parameters
23	Blake et al [23]	Increase PA	SMS and email	Automatic	Knowledge-based	TPB	Motivational content	PA profile and BCT

								parameters
24	Coolbaugh et al [24]	Increase PA	Visual feedback from activity monitor based on prescribed PA and website	Automatic	Knowledge-based	American College of Sports Medicine training progression guidelines for sedentary low-risk adults	Goal recommendation	PA profile
25	Hargreaves et al [25]	Increase PA	Website and message	Automatic	Knowledge-based	PA guideline	Goal recommendation and motivational content	PA profile, demographics, medical data, and BCT parameters

26	Williams et al [26]	Increase PA	Website and email	Automatic	Knowledge-based	Several clinical guidelines	Goal and activity recommendation	PA profile and medical data
27	Kwasnicka et al [27]	Increase PA	Website and SMS	Automatic	Knowledge-based	BCT used	Educational and motivational content	PA profile, demographics, BCT parameters, and contextual information
28	Janols et al [28]	Increase PA	Website and mobile app	Automatic	Knowledge-based	Activity Theory and SDT	Motivational content	PA profile, demographics, medical data, BCT parameters, and contextual information

29	Ali et al [29]	Increase PA	Mobile app	Automatic	Knowledge-based	Center for Disease Control and Prevention guidelines	Goal and Activity recommendation and educational content	PA profile, demographics, and contextual information
30	Mistry et al [30]	Increase PA	SMS and email	Automatic	Knowledge-based	TPB	Motivational content and intervention timing	PA profile, demographics, BCT parameters, and contextual information
31	Peels et al [31]	Increase PA	Website, email, and print	Automatic	Knowledge-based	I-Change Model, TTM, HAPA, Self-regulation Theor	Activity recommendation and educational and motivational content	PA profile, demographics, BCT parameters, and contextual information

						y		
32	Klein et al [32]	Increase PA	Mobile app and website	Automatic	Knowledge-based system	SCT and TTM	Goal and activity recommendation and educational and motivational content	PA profile, demographics, BCT parameters, and contextual information
33	Ammann et al [33]	Increase PA	Website	Automatic	Knowledge-based	TPB and TTM	Educational and motivational content	PA profile, demographics, and BCT parameters
34	Pyky et al [34]	Increase PA	Website	Automatic	Knowledge-based	TTM	Motivational content	PA profile, demographics, BCT parameters, and contextual

								information
35	Varadharajan et al [35]	Increase PA	Mobile app	Automatic	h	In association with behavioral scientists	Goal and fitness partner recommendation and motivational content	PA profile, demographics, BCT parameters, and contextual information
36	Codreanu et al [36]	Increase PA	Kinect	Semiautomated	Knowledge-based system	—	Goal and activity recommendation	PA profile, demographics, medical data, and contextual information
37	Marsaux et al [37]	Increase PA	Website and PDF report	Semiautomated	Machine learning : decision trees and expert	Protection Motivation Theory	Activity recommendation and educational	PA profile, demographics, and medical

							content	data
38	Alley et al [38]	Increase PA	Website	Semiautomatic	Knowledge-based	TPB and Elaboration Likelihood Model	Goal recommendation and motivational and educational content	PA profile, demographics, BCT parameters, and contextual information
39	Mitchell et al [39]	Increase PA	Website, telephone calls	Semiautomatic	Knowledge-based	SCT and SDT	Goal recommendation	PA profile, medical data, and BCT parameters
40	Oosterom-Calo et al [40]	Increase PA+medical adherence	h	Automatic	Knowledge-based system	Principles of TPB and SCT	Educational and motivational content	BCT parameters
41	De Cocker et al [41]	Reduce workplace sitting time : increase	Website	Automatic	Knowledge-based	SDT, TPB, and self-regulat	Activity recommendation and educatio	PA profile, demographics, and

		PA				ion theory	nal and motivational content	BCT parameters
42	Triantafyllidis et al [42]	Rehabilitation	Kinect	Automatic	Knowledge-based	Behavioral change wheel and multiple clinical guidelines	Goal and activity recommendation and motivational content if required	PA profile and medical data
43	Dobrican et al [43]	Rehabilitation	Smartwatch+smartphone app	Semiautomated	Knowledge-based system and doctor recommended target range	—	Goal recommendation	PA profile, demographics, and medical data
44	Hales et al [44]	Weight loss	Mobile app	Automatic	Data-driven recommendation system	SCT	Fitness partner recommendation who provides motivational message	PA profile, demographics, and BCT parameters

45	Martin et al [45]	Weight loss	Mobile app	Semiautomated	Manual and knowledge-based system	Principle of TPB and Theory of reasoned action	Goal recommendation and educational content	PA profile, demographics, and contextual information
46	Spark et al [46]	Weight loss, healthy lifestyle : diet and exercise	SMS	Semiautomated	Knowledge-based	SCT	Goal and activity recommendation, motivational content, and intervention timing	PA profile, demographics, and contextual information
47	Kattelmann et al [47]	Weight management	Website and email	Automatic	Knowledge-based	Stage of Change model	Motivational content	PA profile and BCT parameters
48	Partridge et al [48]	Weight management	SMS and call	Semiautomated	Knowledge-based	Process of change and SCT	Motivational content	Demographics and BCT parameters

49	Walthouwer et al [49]	Weight management :obesity prevention	Website	Automatic	Knowledge-based	TPB, SCT, TTM, and I-Change Model	Educational content	PA profile, demographics, and BCT parameters

[a]TPB: Theory of Planned Behavior.

[b]TTM: Transtheoretical Model.

[c]PA: physical activity.

[d]BCT: behavior change technique.

[e]HAPA: health action process approach.

[f]API: Application programming interface

[g]SCT: Social Cognitive Theory.

[h]: Unclear.

[i]SMS: short messaging service.

[j]RNN: recurrent neural networks.

[k]SDT: self-determination theory.

References:

1. Vandelanotte C, Short C, Plotnikoff RC, Hooker C, Canoy D, Rebar A, et al. TaylorActive - Examining the effectiveness of web-based personally-tailored videos to increase physical activity: a randomised controlled trial protocol. BMC Public Health. BMC Public Health; 2015;15(1):1–14. PMID: 26438225.
2. Ahire SB, Khanuja HK. A Personalized Framework for Health Care Recommendation. 2015 Int Conf Comput Commun Control Autom. IEEE; 2015. p. 442–445. DOI: 10.1109/ICCUBEA.2015.92.
3. Mukhtar H. Using Persuasive Recommendations in Wellness Applications based upon User Activities. Int J Adv Comput Sci Appl. 2016;7(8):349–356. DOI: 10.14569/IJACSA.2016.070847.
4. Tseng JCC, Lin B-HH, Lin Y-FF, Tseng VS, Day M-LL, Wang S-CC, et al. An interactive healthcare system with personalized diet and exercise guideline recommendation. TAAI

2015 - 2015 Conf Technol Appl Artif Intell. IEEE; 2016;525–532. DOI: 10.1109/TAAI.2015.7407106.

5. Storm V, Dörenkämper J, Reinwand DA, Wienert J, De Vries H, Lippke S. Effectiveness of a Web-Based Computer-Tailored Multiple-Lifestyle Intervention for People Interested in Reducing their Cardiovascular Risk: A Randomized Controlled Trial. J Med Internet Res. 2016;18(4):e78. PMID: 27068880.

6. Schulz DN, Kremers SPJJ, Vandelanotte C, van Adrichem MJG, Schneider F, Candel MJJMJM, et al. Effects of a web-based tailored multiple-lifestyle intervention for adults: a two-year randomized controlled trial comparing sequential and simultaneous delivery modes. J Med Internet Res. Canada; 2014;16(1):e26. PMID: 24472854.

7. Hermens H, op den Akker H, Tabak M, Wijsman J, Vollenbroek M. Personalized Coaching Systems to support healthy behavior in people with chronic conditions. J Electromyogr Kinesiol. Elsevier Ltd; 2014;24(6):815–26. PMID: 25455254.

8. Lee MK, Park H-AA, Yun YH, Chang YJ. Development and formative evaluation of a web-based self-management exercise and diet intervention program with tailored motivation and action planning for cancer survivors. JMIR Res Protoc. Canada; 2013;2(1):e11. PMID: 23612029.

9. Fahim M, Idris M, Ali R, Nugent C, Kang B, Huh E-NN, et al. ATHENA: A personalized platform to promote an active lifestyle and wellbeing based on physical, mental and social health primitives. Sensors (Switzerland). 2014;14(5):9313–9329. PMID: 24859031.

10. Dharia S, Jain V, Patel J, Vora J, Yamauchi R, Eirinaki M, et al. PRO-Fit: Exercise with friends. Proc 2016 IEEE/ACM Int Conf Adv Soc Networks Anal Mining, ASONAM 2016. IEEE; 2016;1430–1433. DOI: 10.1109/ASONAM.2016.7752437.

11. Rabbi M, Aung MH, Zhang M, Choudhury T. MyBehavior: automatic personalized health feedback from user behaviors and preferences using smartphones. Ubicomp ’15, Sept 7–11, 2015, Osaka, Japan. 2015;707–718. PMID: 25977197.

12. Twardowski B, Ryzko D. IoT and Context-Aware Mobile Recommendations Using Multi-agent Systems. 2015 IEEE/WIC/ACM Int Conf Web Intell Intell Agent Technol. IEEE; 2015. p. 33–40. DOI: 10.1109/WI-IAT.2015.120.

13. Yom-Tov E, Feraru G, Kozdoba M, Mannor S, Tennenholtz M, Hochberg I. Encouraging physical activity in patients with diabetes: Intervention using a reinforcement learning system. J Med Internet Res. 2017;19(10):e338. PMID: 29017988.

14. Lim C-G, Kim ZM, Choi H-J. Developing a Mobile Wellness Management System for Healthy Lifestyle by Analyzing Daily Living Activities. Stud Health Technol Inform. Netherlands; 2017;245:146–150. PMID: 29295070.

15. Cook TL, De Bourdeaudhuij I, Maes L, Haerens L, Grammatikaki E, Widhalm K, et al. Moderators of the effectiveness of a web-based tailored intervention promoting physical activity in adolescents: the HELENA Activ-O-Meter. J Sch Health. 2014;84(4):256–66. PMID: 24617909.

16. Larsen B, Benitez T, Cano M, Dunsiger SS, Marcus BH, Mendoza-Vasconez A, et al.

Web-Based Physical Activity Intervention for Latina Adolescents: Feasibility, Acceptability, and Potential Efficacy of the Niñas Saludables Study. J Med Internet Res. Journal of Medical Internet Research; 2018;20(5):e170. PMID: 29743151.

17. Short CE, Rebar A, James EL, Duncan MJ, Courneya KS, Plotnikoff RC, et al. How do different delivery schedules of tailored web-based physical activity advice for breast cancer survivors influence intervention use and efficacy? J Cancer Surviv. Journal of Cancer Survivorship; 2017;11(1):80–91. PMID: 27498099.

18. Boudreau FF, Walthouwer MJL, De Vries H, Dagenais GR, Turbide G, Bourlaud A-SS, et al. Rationale, design and baseline characteristics of a randomized controlled trial of a web-based computer-tailored physical activity intervention for adults from Quebec City. BMC Public Health. England: BMC Public Health; 2015;15(1):1038. PMID: 26453041.

19. Moreau M, Gagnon M-P, Boudreau FF. Development of a fully automated, web-based, tailored intervention promoting regular physical activity among insufficiently active adults with type 2 diabetes: integrating the I-change model, self-determination theory, and motivational interviewing compon. JMIR Res Protoc. Canada; 2015;4(1):e25. PMID: 25691346.

20. Rajanna V, Behera DJ, Goldberg D, Hammond T. Step Up Life: A Context Aware Health Assistant. HealthGIS'14. 2014;21–30. DOI: 10.1145/2676629.2676636.

21. Irvine AB, Gelatt VA, Seeley JR, Macfarlane P, Gau JM. Web-based intervention to promote physical activity by sedentary older adults: randomized controlled trial. Ajzen Ajzen, Allison, Chodzko-Zajko, Armitage, Bandura, Bandura, Baranowski, Beauchamp, Blissmer, Bock, Booth, Bowen, Bowles, Calfas, Carlson, Christensen, Christensen, Cohen, Conn, Conn, Cress, Danaher, Davies, DiBrezzo, Doshi, Duncan, Eysenbach, Fillen A, editor. J Med Internet Res. Irvine, A. Blair: ORCAS, 260 E. 11th Avenue, Eugene, OR, US, 97401, birvine@orcasinc.com: Gunther Eysenbach; 2013;15(2):e19. PMID: 23470322.

22. Friederichs SAHH, Oenema A, Bolman C, Lechner L. Motivational interviewing and self-determination theory in a web-based computer tailored physical activity intervention: A randomized controlled trial. Psychol Heal. Routledge; 2016;31(8):907–930. PMID: 26849996.

23. Blake H, Suggs LS, Coman E, Aguirre L, Batt ME. Active8! Technology-Based Intervention to Promote Physical Activity in Hospital Employees. Am J Heal Promot. 2017;31(2):109–118. PMID: 26559712.

24. Coolbaugh CL, Raymond SC, Hawkins DA, Raymond Jr SC, Hawkins DA. Feasibility of a dynamic web guidance approach for personalized physical activity prescription based on daily information from wearable technology. JMIR Res Protoc. 2015;4(2):e67. PMID: 26043793.

25. Hargreaves EA, Mutrie N, Fleming JD. A Web-Based Intervention to Encourage Walking (StepWise): Pilot Randomized Controlled Trial. JMIR Res Protoc. Canada; 2016;5(1):e14. PMID: 26810251.

26. Williams QI, Gunn AH, Beaulieu JE, Benas BC, Buley B, Callahan LF, et al. Physical

therapy vs. internet-based exercise training (PATH-IN) for patients with knee osteoarthritis: study protocol of a randomized controlled trial. BMC Musculoskelet Disord. BMC Musculoskeletal Disorders; 2015;16(1):264. PMID: 26416025.

27. Kwasnicka D, Vandelanotte C, Rebar A, Gardner B, Short C, Duncan M, et al. Comparing motivational, self-regulatory and habitual processes in a computer-tailored physical activity intervention in hospital employees - protocol for the PATHS randomised controlled trial. BMC Public Health. BMC Public Health; 2017;17(1):518. PMID: 28549476.

28. Janols R, Guerrero E, Lindgren H. A pilot study on personalised coaching to increase older adults' physical and social activities [Internet]. Adv Intell Syst Comput. 2017. p. 140–148. DOI: 10.1007/978-3-319-61118-1_18.

29. Ali R, Afzal M, Hussain M, Ali M, Siddiqi MH, Lee S, et al. Multimodal hybrid reasoning methodology for personalized wellbeing services. Comput Biol Med. Elsevier; 2016;69:10–28. PMID: 26705863.

30. Mistry CD, Sweet SN, Rhodes RE, Latimer-Cheung AE. Text2Plan: Exploring changes in the quantity and quality of action plans and physical activity in a text messaging intervention. Psychol Health. England: Routledge; 2015;30(7):839–56. PMID: 25659571.

31. Peels DA, Hoogenveen RR, Feenstra TL, Golsteijn RH, Bolman C, Mudde AN, et al. Long-term health outcomes and cost-effectiveness of a computer-tailored physical activity intervention among people aged over fifty: modelling the results of a randomized controlled trial. BMC Public Health. England; 2014;14(1):1099. PMID: 25342517.

32. Klein MCAA, Manzoor A, Mollee JS. Active2Gether: A personalized m-health intervention to encourage physical activity. Sensors (Switzerland). 2017;17(6):1–16. PMID: 28629178.

33. Ammann R, Vandelanotte C, de Vries H, Mummery WK. Can a Website-Delivered Computer-Tailored Physical Activity Intervention Be Acceptable, Usable, and Effective for Older People? Health Educ Behav. 2013;40(2):160–170. PMID: 23077157.

34. Pyky R, Koivumaa-Honkanen H, Leinonen A-MM, Ahola R, Hirvonen N, Enwald H, et al. Effect of tailored, gamified, mobile physical activity intervention on life satisfaction and self-rated health in young adolescent men: A population-based, randomized controlled trial (MOPO study). Comput Human Behav. 2017;72:13–22. DOI: 10.1016/j.chb.2017.02.032.

35. Varadharajan V, Kannav V, Pasala A, Kannav, Pasala. Sensor based coaching for a fit and healthy society. Int Symp Technol Soc Proc. IEEE; 2016;2016–Decem(October):81–85. DOI: 10.1109/ISTAS.2016.7764275.

36. Codreanu IA, Florea AM. A Proposed Serious Game Architecture to Self-Management HealthCare for Older Adults. Proc - 17th Int Symp Symb Numer Algorithms Sci Comput SYNASC 2015. IEEE; 2016;437–440. DOI: 10.1109/SYNASC.2015.71.

37. Marsaux CFMM, Celis-Morales C, Livingstone KM, Fallaize R, Kolossa S, Hallmann J, et al. Changes in physical activity following a genetic-based internet-delivered

personalized intervention: Randomized controlled trial (Food4Me). J Med Internet Res. Canada; 2016;18(2):1–16. PMID: 26851191.

38. Alley S, Jennings C, Plotnikoff RC, Vandelanotte C. Web-based video-coaching to assist an automated computer-tailored physical activity intervention for inactive adults: A randomized controlled trial. J Med Internet Res. 2016;18(8):1–16. PMID: 27520283.

39. Mitchell BL, Smith AE, Rowlands A V., Fraysse F, Parfitt G, Lewis NR, et al. Promoting physical activity in rural Australian adults using an online intervention. J Sci Med Sport. Sports Medicine Australia; 2018; PMID: 30031747.

40. Oosterom-Calo R, Te Velde SJ, Stut W, Brug J. Development of Motivate4Change Using the Intervention Mapping Protocol: An Interactive Technology Physical Activity and Medication Adherence Promotion Program for Hospitalized Heart Failure Patients. JMIR Res Protoc. 2015;4(3):e88. PMID: 26195072.

41. De Cocker K, De Bourdeaudhuij I, Cardon G, Vandelanotte C. The Effectiveness of a Web-Based Computer-Tailored Intervention on Workplace Sitting: A Randomized Controlled Trial. J Med Internet Res. 2016;18(5):e96. PMID: 27245789.

42. Triantafyllidis A, Filos D, Buys R, Claes J, Cornelissen V, Kouidi E, et al. Computerized decision support for beneficial home-based exercise rehabilitation in patients with cardiovascular disease. Comput Methods Programs Biomed. 2018;162(May):1–10. PMID: 29903475.

43. Dobrican RA, Zampunieris D. A Proactive Solution, using Wearable and Mobile Applications, for Closing the Gap between the Rehabilitation Team and Cardiac Patients. Proc - 2016 IEEE Int Conf Healthc Informatics, ICHI 2016. IEEE; 2016. p. 146–155. DOI: 10.1109/ICHI.2016.23.

44. Hales S, Turner-McGrievy G, Fahim A, Freix A, Wilcox S, Davis RE, et al. A Mixed-Methods Approach to the Development, Refinement, and Pilot Testing of Social Networks for Improving Healthy Behaviors. JMIR Hum Factors. 2016;3(1):e8. PMID: 27026535.

45. Martin CK, Gilmore LA, Apolzan JW, Myers CA, Thomas DM, Redman LM. Smartloss: A Personalized Mobile Health Intervention for Weight Management and Health Promotion. JMIR mHealth uHealth. Canada; 2016;4(1):e18. PMID: 26983937.

46. Spark LC, Fjeldsoe BS, Eakin EG, Reeves MM. Efficacy of a Text Message-Delivered Extended Contact Intervention on Maintenance of Weight Loss, Physical Activity, and Dietary Behavior Change. JMIR mHealth uHealth. 2015;3(3):e88. PMID: 26373696.

47. Kattelmann KK, White AA, Greene GW, Byrd-Bredbenner C, Hoerr SL, Horacek TM, et al. Development of Young Adults Eating and Active for Health (YEAH) internet-based intervention via a community-based participatory research model. J Nutr Educ Behav. Elsevier Inc.; 2014;46(2):S10-25. PMID: 24456834.

48. Partridge SR, McGeechan K, Hebden L, Balestracci K, Wong AT, Denney-Wilson E, et al. Effectiveness of a mHealth Lifestyle Program With Telephone Support (TXT2BFiT) to Prevent Unhealthy Weight Gain in Young Adults: Randomized Controlled Trial. JMIR mHealth uHealth. Canada; 2015;3(2):e66. PMID: 26076688.

49. Walthouwer MJL, Oenema A, Soetens K, Lechner L, De Vries H. Systematic development of a text-driven and a video-driven web-based computer-tailored obesity prevention intervention. BMC Public Health. England; 2013;13(1):978. PMID: 24138937.
