# Supplementary material for: User Models for Personalized Physical Activity Interventions: Scoping Review
Source: JMIR Mhealth Uhealth. 2019 Jan 16;7(1):e11098. doi: 10.2196/11098 (PMC6352015; doi:10.2196/11098)
Supplement: Multimedia Appendix 2 [file mhealth_v7i1e11098_app2.pdf]

This is a Multimedia Appendix to a full manuscript published in the J Med Internet Res. For full copyright and citation information see http://dx.doi.org/10.2196/11098.

Table: User Model Parameters

Serial number	Paper reference	PA[a] profile	Demographics	Medical data	BCT[b] parameters	Contextual information
1	Vandelanotte et al [1]	Activity monitor data, sitting time, and history of feedback	age, gender, education, BMI[c], employment, income, postcode, and address	Presence or absence of depression, anxiety, and stress; sleep behavior and duration[d]	Subjective PA, motivation, action planning, and goal setting	Quality of life, perceived neighborhood environment[d], learning style, and delivery mode preference
2	Ahire et al [2]	Lifestyle	Age and weight	Medical condition and allergies	—	Preferences
3	Mukhtar [3]	User activities type, duration and difficulty level, and history of activities	Age and gender	Medications, chronic conditions, disabilities, and medical history	—	User preferences on exercise type and routine and diet and social interests
4	Tseng et al [4]	User physiological value and	—	Health assessment reports	—	—

		history of recommendations				
5	Storm et al [5]	Self-reported PA and goal (static)	Gender, country, marital status, BMI, year of birth, employment, and education	—	Self-efficacy, planning, habit strength, stage, intention, and risk perception	—
6	Schulz et al [6]	PA level	Age, gender, height, weight, marital status, religious background, ethnicity, educational level, current work status, and income	Presence of high blood pressure, diabetes, or cancer	Attitude, barriers, self-efficacy, intention, and social support	Quality of life, smoking, alcohol consumption, and fruits and vegetable intake
7	Hermens et al [7]	PA (in energy expenditure), history of	Age, height, and weight	—	Stage of change and self-efficacy	Current time, day weather, and temperature

		sent messages, and user goal				
8	Lee et al [8]	Type, intensity, duration, PA level, and history of exercise	Weight, BMI, and age	—	Barriers, stage of change, and motivational readiness	Preference of exercise and tiredness
9	Fahim et al [9]	User activities and history of activities	Age and gender (height and weight from case study)	—	—	Social profile, emotion, preferred activity, food, and music
10	Dharia et al [10]	Accelerometer data, calories, history of activity	Age and gender	—	—	Location, activity preferences, and calendar availability
11	Rabbi et al [11]	Accelerometer data, history of activity, MET[e] and calorie for each activity, and weekly weight goal	Age, gender, height, and weight	—	—	User preference for suggestion provided and location

12	Twardowski et al [12]	Fitness level, current activity, and next predicted activity	Weight	Health care record and pulse	—	Calendar
13	Yom-Tov et al [13]	Previous day PA, cumulative number of minutes of PA in week, fraction of activity goal, fraction versus expected at this point in the week, history of messages sent, and number of days since feedback message sent	Age and gender	—	—	—
14	Lim et al [14]	HR[f], Step, calorie, history of recommendations	—	—	—	Location, mood, User feedback on recommendations

15	Cook et al [15]	PA level, minutes of PA per day, and minutes of MVPA[g]	Age and gender	—	Attitudes, self-efficacy, social support, perceived benefits, perceived barriers, and environment	—
16	Larsen et al [16]	Frequency, duration, and intensity of MVPA	Height, weight, income, marital status, and family size	Resting blood pressure	Motivational stage for MVPA change, MVPA change strategies, self-efficacy, decisional balance, family influence and support, peer influence and support, enjoyment for activity, and environmental access	—

17	Short et al [17]	Minutes of MVPA per week and resistance training score	Height, weight, and BMI	Stage of breast cancer, time since treatment, current menopausal status, and number of comorbidities	Intention, barrier, outcome expectations, social support, and self-efficacy	Location and fatigue
18	Boudreau et al [18]	Baseline PA level and minutes of PA per week	Age, gender, marital status, occupation, and education	—	Intention, self-efficacy, social influence, attitude, perceived benefits, and barriers	—
19	Moreau et al [19]	Number of times/week and minutes/time for vigorous, moderate, and low PA	Height, weight, age, gender, occupation, origins, and marital status	—	Intention, attitude, self-efficacy, motivation, and confidence	—
20	Rajanna et al [20]	Activity history	Age	—	—	Location, time of day, weather, and calendar

21	Irvine et al [21]	Self-reported PA (frequency and duration), activity plan, and goal	—	—	Intention, barriers, self-efficacy, stage of change, and motivation	—
22	Friederichs et al [22]	PA level	Age, gender, and BMI	—	Efficacy, confidence, and intention	—
23	Blake et al [23]	Minutes of PA	—	—	Attitude, subjective norm, perceived behavior control, intention, and behavior	—
24	Coolbaugh et al [24]	PA duration, HR, resting HR, accelerometer data, and goal (system generated adaptive)	—	—	—	—
25	Hargreaves et al [25]	Step count	Height, weight, and BMI	Medication for blood pressure or cholesterol, resting HR, blood	Motivation, intention, self-efficacy, and barriers	—

				pressure, glucose, cholesterol, HDL[h], and triglycerides		
26	Williams et al [26]	Self-reported activity level, exercise history, and daily activities	—	Stiffness and pain	—	—
27	Kwasnicka et al [27]	PA minutes, and goal	Age, sex, date of birth, height, weight, handedness, marital status, ethnicity, education, household income, post code, number of hours and days worked per week, work	Sleep quality and duration[d]	Knowledge, self-efficacy, attitude, perceived behavioral control, intentions, and outcome expectations	—

			level, salary brackets associated with each employment level, and type of work undertaken			
28	Janols et al [28]	Daily and weekly habits	Gender, age, social factors, and living condition	Pain and sleeping conditions	Motivational info available with each category of activity	Priority of activities and preference
29	Ali et al [29]	Accelerometer data, duration of each activity, calories, and recommended goal (in METs)	Age, gender, height, and weight	—	—	Preferred activities
30	Mistry et al [30]	Quantity and duration of MVPA and goal	Age, gender, minority status, education, employment,	—	Perceived barriers and benefits	Preference of day and time

			household income and marital status			
31	Peels et al [31]	Minutes of PA	Gender and age	—	Stage of change, awareness, attitude, knowledge, self-efficacy, motivation, and intention	Location and neighborhood
32	Klein et al [32]	Steps and stairs climbed	Occupation	—	Skills, barriers, outcome expectations, subjective PA, and stage of change	Current location, significant locations, current day, time, weather, and social profile
33	Ammann et al [33]	PA level measured using the Active Australia Survey	Age, height, weight, education, employment, and BMI	—	Attitudes, self-efficacy, intention, benefits, and barriers	—
34	Pyky et al [34]	Accumulated daily MVPA time and daily	Height, weight, socioeconomic	—	Stage of change, life satisfaction, and self-	Social profile

		sitting time	status, and family income		rated health	
35	Varadharajan et al [35]	Accelerometer data, speed, distance, and HR	Age, height, and weight	—	Motivation	Mood and social profile
36	Codreanu et al [36]	Execution speed, difficulty level, target zone for HR, pulse, time, and number of exercises	Age and weight	Symptoms	—	“mood temperature factor”
37	Marsaux et al [37]	Daily PA level, activity energy expenditure (AEE), and time spent in different PA intensities	BMI and waist measurements	Blood total cholesterol, L2 and L3, fat mass- and obesity-associated gene, and FTO gene	—	—

38	Alley et al [38]	PA level, number of activity sessions per week, and goal	BMI and age	—	Perceived benefits, barriers, self-efficacy, and attitude	Preference of video calling service, possession of a garden, distance to places regularly visited, working status, length of lunch break, facilities at work, and activity level of people around
39	Mitchell et al [39]	Step count, PA monitoring at baseline, time in LPA[i] and MVPA, average signal vector magnitude value, and average daily	—	Anthropometry, blood pressure, and blood chemistry	Perceived barriers, rate of perceived exertion, and affective state	—

		sedentary time				
40	Oosterom-Calo et al [40]	—	—	—	Self-efficacy and knowledge	—
41	De Cocker et al [41]	ActivPal measurements, PA from questionnaires, personalized action plan with goal, level of sitting time in 5 domains, and frequency of interruptions in prolonged sitting	Gender, age, education, occupation, height, weight, and work hours	—	User attitudes, self-efficacy, social support, knowledge, intentions, and perceived benefits and barriers related to reducing or interrupting sitting	—
42	Triantafyllidis et al [42]	HR, repetitions set, accuracy of exercise, history log	—	medical condition, pain, and blood pressure	—	—
43	Dobrican et al [43]	HR, training zone, duration,	Age	Anaerobic threshold and status	—	—

		frequency, and intensity of training session		of whether patient under treatment		
44	Hales et al [44]	Minutes of intentional PA and total calories	Weight, gender, race, BMI, education, and marital status	—	Self-efficacy, and personality characteristic[d]	—
45	Martin et al [45]	Steps, “zone of adherence,” and activity	Age, sex, weight, and height	—	—	Preference of calorie intake
46	Spark et al [46]	Minutes per day MVPA, Average MVPA minutes, and goal	Weight	—	—	Preference for content, timing and frequency of text messages, and self-nominated reward for reaching goal
47	Kattelmann et al [47]	PA, food, and goal	—	—	Contemplators, precontemplators, and stage	—

48	Partridge et al [48]	—	Age and gender	—	Participant’s baseline stage of change and perceived benefits and barriers	—
49	Walthouwer et al [49]	Diet and exercise pattern	Weight and BMI	—	Attitude, self-efficacy, and social influence beliefs regarding PA and diet	—

[a]PA: physical activity.

[b]BCT: behavior change technique.

[c]BMI: body mass index.

[d]Measured but unclear if used for personalization.

[e]MET: metabolic equivalent.

[f]HR: heart rate.

[g]MVPA: moderately vigorous physical activity.

[h]HDL: high-density lipoprotein.

[i]LPA: light physical activity.

— : indicates no parameters present in the category

References:

1. Vandelanotte C, Short C, Plotnikoff RC, Hooker C, Canoy D, Rebar A, et al. TaylorActive - Examining the effectiveness of web-based personally-tailored videos to increase physical activity: a randomised controlled trial protocol. BMC Public Health. BMC Public Health; 2015;15(1):1–14. PMID: 26438225.

2. Ahire SB, Khanuja HK. A Personalized Framework for Health Care Recommendation. 2015 Int Conf Comput Commun Control Autom. IEEE; 2015. p. 442–445. DOI: 10.1109/ICCUBEA.2015.92.

3. Mukhtar H. Using Persuasive Recommendations in Wellness Applications based upon User Activities. Int J Adv Comput Sci Appl. 2016;7(8):349–356. DOI: 10.14569/IJACSA.2016.070847.

4. Tseng JCC, Lin B-HH, Lin Y-FF, Tseng VS, Day M-LL, Wang S-CC, et al. An interactive healthcare system with personalized diet and exercise guideline recommendation. TAAI 2015 - 2015 Conf Technol Appl Artif Intell. IEEE; 2016;525–532. DOI: 10.1109/TAAI.2015.7407106.

5. Storm V, Dörenkämper J, Reinwand DA, Wienert J, De Vries H, Lippke S. Effectiveness of a Web-Based Computer-Tailored Multiple-Lifestyle Intervention for People Interested in Reducing their Cardiovascular Risk: A Randomized Controlled Trial. J Med Internet Res. 2016;18(4):e78. PMID: 27068880.

6. Schulz DN, Kremers SPJJ, Vandelanotte C, van Adrichem MJG, Schneider F, Candel MJJMJM, et al. Effects of a web-based tailored multiple-lifestyle intervention for adults: a two-year randomized controlled trial comparing sequential and simultaneous delivery modes. J Med Internet Res. Canada; 2014;16(1):e26. PMID: 24472854.

7. Hermens H, op den Akker H, Tabak M, Wijsman J, Vollenbroek M. Personalized Coaching Systems to support healthy behavior in people with chronic conditions. J Electromyogr Kinesiol. Elsevier Ltd; 2014;24(6):815–26. PMID: 25455254.

8. Lee MK, Park H-AA, Yun YH, Chang YJ. Development and formative evaluation of a web-based self-management exercise and diet intervention program with tailored motivation and action planning for cancer survivors. JMIR Res Protoc. Canada; 2013;2(1):e11. PMID: 23612029.

9. Fahim M, Idris M, Ali R, Nugent C, Kang B, Huh E-NN, et al. ATHENA: A personalized platform to promote an active lifestyle and wellbeing based on physical, mental and social health primitives. Sensors (Switzerland). 2014;14(5):9313–9329. PMID: 24859031.

10. Dharia S, Jain V, Patel J, Vora J, Yamauchi R, Eirinaki M, et al. PRO-Fit: Exercise with friends. Proc 2016 IEEE/ACM Int Conf Adv Soc Networks Anal Mining, ASONAM 2016. IEEE; 2016;1430–1433. DOI: 10.1109/ASONAM.2016.7752437.

11. Rabbi M, Aung MH, Zhang M, Choudhury T. MyBehavior: automatic personalized health feedback from user behaviors and preferences using smartphones. Ubicomp ’15, Sept 7–11, 2015, Osaka, Japan. 2015;707–718. PMID: 25977197.

12. Twardowski B, Ryzko D. IoT and Context-Aware Mobile Recommendations Using Multi-agent Systems. 2015 IEEE/WIC/ACM Int Conf Web Intell Intell Agent Technol. IEEE; 2015. p. 33–40. DOI: 10.1109/WI-IAT.2015.120.

13. Yom-Tov E, Feraru G, Kozdoba M, Mannor S, Tennenholtz M, Hochberg I. Encouraging physical activity in patients with diabetes: Intervention using a reinforcement learning system. J Med Internet Res. 2017;19(10):e338. PMID: 29017988.

14. Lim C-G, Kim ZM, Choi H-J. Developing a Mobile Wellness Management System for Healthy Lifestyle by Analyzing Daily Living Activities. Stud Health Technol Inform. Netherlands; 2017;245:146–150. PMID: 29295070.

15. Cook TL, De Bourdeaudhuij I, Maes L, Haerens L, Grammatikaki E, Widhalm K, et al. Moderators of the effectiveness of a web-based tailored intervention promoting physical activity in adolescents: the HELENA Activ-O-Meter. J Sch Health. 2014;84(4):256–66. PMID: 24617909.

16. Larsen B, Benitez T, Cano M, Dunsiger SS, Marcus BH, Mendoza-Vasconez A, et al. Web-Based Physical Activity Intervention for Latina Adolescents: Feasibility, Acceptability, and Potential Efficacy of the Niñas Saludables Study. J Med Internet Res. Journal of Medical Internet Research; 2018;20(5):e170. PMID: 29743151.

17. Short CE, Rebar A, James EL, Duncan MJ, Courneya KS, Plotnikoff RC, et al. How do different delivery schedules of tailored web-based physical activity advice for breast cancer survivors influence intervention use and efficacy? J Cancer Surviv. Journal of Cancer Survivorship; 2017;11(1):80–91. PMID: 27498099.

18. Boudreau FF, Walthouwer MJL, De Vries H, Dagenais GR, Turbide G, Bourlaud A-SS, et al. Rationale, design and baseline characteristics of a randomized controlled trial of a web-based computer-tailored physical activity intervention for adults from Quebec City. BMC Public Health. England: BMC Public Health; 2015;15(1):1038. PMID: 26453041.

19. Moreau M, Gagnon M-P, Boudreau FF. Development of a fully automated, web-based, tailored intervention promoting regular physical activity among insufficiently active adults with type 2 diabetes: integrating the I-change model, self-determination theory, and motivational interviewing compon. JMIR Res Protoc. Canada; 2015;4(1):e25. PMID: 25691346.

20. Rajanna V, Behera DJ, Goldberg D, Hammond T. Step Up Life: A Context Aware Health Assistant. HealthGIS'14. 2014;21–30. DOI: 10.1145/2676629.2676636.

21. Irvine AB, Gelatt VA, Seeley JR, Macfarlane P, Gau JM. Web-based intervention to promote physical activity by sedentary older adults: randomized controlled trial. Ajzen Ajzen, Allison, Chodzko-Zajko, Armitage, Bandura, Bandura, Baranowski, Beauchamp, Blissmer, Bock, Booth, Bowen, Bowles, Calfas, Carlson, Christensen, Christensen, Cohen, Conn, Conn, Cress, Danaher, Davies, DiBrezzo, Doshi, Duncan, Eysenbach, Fillen A, editor. J Med Internet Res. Irvine, A. Blair: ORCAS, 260 E. 11th Avenue, Eugene, OR, US, 97401, birvine@orcasinc.com: Gunther Eysenbach; 2013;15(2):e19. PMID: 23470322.

22. Friederichs SAHH, Oenema A, Bolman C, Lechner L. Motivational interviewing and self-determination theory in a web-based computer tailored physical activity intervention: A randomized controlled trial. Psychol Heal. Routledge; 2016;31(8):907–930. PMID: 26849996.

23. Blake H, Suggs LS, Coman E, Aguirre L, Batt ME. Active8! Technology-Based Intervention to Promote Physical Activity in Hospital Employees. Am J Heal Promot. 2017;31(2):109–118. PMID: 26559712.

24. Coolbaugh CL, Raymond SC, Hawkins DA, Raymond Jr SC, Hawkins DA. Feasibility of a dynamic web guidance approach for personalized physical activity prescription based on daily information from wearable technology. JMIR Res Protoc. 2015;4(2):e67. PMID:

26043793.

25. Hargreaves EA, Mutrie N, Fleming JD. A Web-Based Intervention to Encourage Walking (StepWise): Pilot Randomized Controlled Trial. JMIR Res Protoc. Canada; 2016;5(1):e14. PMID: 26810251.

26. Williams QI, Gunn AH, Beaulieu JE, Benas BC, Buley B, Callahan LF, et al. Physical therapy vs. internet-based exercise training (PATH-IN) for patients with knee osteoarthritis: study protocol of a randomized controlled trial. BMC Musculoskelet Disord. BMC Musculoskeletal Disorders; 2015;16(1):264. PMID: 26416025.

27. Kwasnicka D, Vandelanotte C, Rebar A, Gardner B, Short C, Duncan M, et al. Comparing motivational, self-regulatory and habitual processes in a computer-tailored physical activity intervention in hospital employees - protocol for the PATHS randomised controlled trial. BMC Public Health. BMC Public Health; 2017;17(1):518. PMID: 28549476.

28. Janols R, Guerrero E, Lindgren H. A pilot study on personalised coaching to increase older adults' physical and social activities [Internet]. Adv Intell Syst Comput. 2017. p. 140–148. DOI: 10.1007/978-3-319-61118-1_18.

29. Ali R, Afzal M, Hussain M, Ali M, Siddiqi MH, Lee S, et al. Multimodal hybrid reasoning methodology for personalized wellbeing services. Comput Biol Med. Elsevier; 2016;69:10–28. PMID: 26705863.

30. Mistry CD, Sweet SN, Rhodes RE, Latimer-Cheung AE. Text2Plan: Exploring changes in the quantity and quality of action plans and physical activity in a text messaging intervention. Psychol Health. England: Routledge; 2015;30(7):839–56. PMID: 25659571.

31. Peels DA, Hoogenveen RR, Feenstra TL, Golsteijn RH, Bolman C, Mudde AN, et al. Long-term health outcomes and cost-effectiveness of a computer-tailored physical activity intervention among people aged over fifty: modelling the results of a randomized controlled trial. BMC Public Health. England; 2014;14(1):1099. PMID: 25342517.

32. Klein MCAA, Manzoor A, Mollee JS. Active2Gether: A personalized m-health intervention to encourage physical activity. Sensors (Switzerland). 2017;17(6):1–16. PMID: 28629178.

33. Ammann R, Vandelanotte C, de Vries H, Mummery WK. Can a Website-Delivered Computer-Tailored Physical Activity Intervention Be Acceptable, Usable, and Effective for Older People? Health Educ Behav. 2013;40(2):160–170. PMID: 23077157.

34. Pyky R, Koivumaa-Honkanen H, Leinonen A-MM, Ahola R, Hirvonen N, Enwald H, et al. Effect of tailored, gamified, mobile physical activity intervention on life satisfaction and self-rated health in young adolescent men: A population-based, randomized controlled trial (MOPO study). Comput Human Behav. 2017;72:13–22. DOI: 10.1016/j.chb.2017.02.032.

35. Varadharajan V, Kannav V, Pasala A, Kannav, Pasala. Sensor based coaching for a fit and healthy society. Int Symp Technol Soc Proc. IEEE; 2016;2016–Decem(October):81–85. DOI: 10.1109/ISTAS.2016.7764275.

36. Codreanu IA, Florea AM. A Proposed Serious Game Architecture to Self-Management HealthCare for Older Adults. Proc - 17th Int Symp Symb Numer Algorithms Sci Comput SYNASC 2015. IEEE; 2016;437–440. DOI: 10.1109/SYNASC.2015.71.

37. Marsaux CFMM, Celis-Morales C, Livingstone KM, Fallaize R, Kolossa S, Hallmann J, et al. Changes in physical activity following a genetic-based internet-delivered personalized intervention: Randomized controlled trial (Food4Me). J Med Internet Res. Canada; 2016;18(2):1–16. PMID: 26851191.

38. Alley S, Jennings C, Plotnikoff RC, Vandelanotte C. Web-based video-coaching to assist an automated computer-tailored physical activity intervention for inactive adults: A randomized controlled trial. J Med Internet Res. 2016;18(8):1–16. PMID: 27520283.

39. Mitchell BL, Smith AE, Rowlands A V., Fraysse F, Parfitt G, Lewis NR, et al. Promoting physical activity in rural Australian adults using an online intervention. J Sci Med Sport. Sports Medicine Australia; 2018; PMID: 30031747.

40. Oosterom-Calo R, Te Velde SJ, Stut W, Brug J. Development of Motivate4Change Using the Intervention Mapping Protocol: An Interactive Technology Physical Activity and Medication Adherence Promotion Program for Hospitalized Heart Failure Patients. JMIR Res Protoc. 2015;4(3):e88. PMID: 26195072.

41. De Cocker K, De Bourdeaudhuij I, Cardon G, Vandelanotte C. The Effectiveness of a Web-Based Computer-Tailored Intervention on Workplace Sitting: A Randomized Controlled Trial. J Med Internet Res. 2016;18(5):e96. PMID: 27245789.

42. Triantafyllidis A, Filos D, Buys R, Claes J, Cornelissen V, Kouidi E, et al. Computerized decision support for beneficial home-based exercise rehabilitation in patients with cardiovascular disease. Comput Methods Programs Biomed. 2018;162(May):1–10. PMID: 29903475.

43. Dobrican RA, Zampunieris D. A Proactive Solution, using Wearable and Mobile Applications, for Closing the Gap between the Rehabilitation Team and Cardiac Patients. Proc - 2016 IEEE Int Conf Healthc Informatics, ICHI 2016. IEEE; 2016. p. 146–155. DOI: 10.1109/ICHI.2016.23.

44. Hales S, Turner-McGrievy G, Fahim A, Freix A, Wilcox S, Davis RE, et al. A Mixed-Methods Approach to the Development, Refinement, and Pilot Testing of Social Networks for Improving Healthy Behaviors. JMIR Hum Factors. 2016;3(1):e8. PMID: 27026535.

45. Martin CK, Gilmore LA, Apolzan JW, Myers CA, Thomas DM, Redman LM. Smartloss: A Personalized Mobile Health Intervention for Weight Management and Health Promotion. JMIR mHealth uHealth. Canada; 2016;4(1):e18. PMID: 26983937.

46. Spark LC, Fjeldsoe BS, Eakin EG, Reeves MM. Efficacy of a Text Message-Delivered Extended Contact Intervention on Maintenance of Weight Loss, Physical Activity, and Dietary Behavior Change. JMIR mHealth uHealth. 2015;3(3):e88. PMID: 26373696.

47. Kattelmann KK, White AA, Greene GW, Byrd-Bredbenner C, Hoerr SL, Horacek TM, et al. Development of Young Adults Eating and Active for Health (YEAH) internet-based intervention via a community-based participatory research model. J Nutr Educ Behav.

Elsevier Inc.; 2014;46(2):S10-25. PMID: 24456834.

48. Partridge SR, McGeechan K, Hebden L, Balestracci K, Wong AT, Denney-Wilson E, et al. Effectiveness of a mHealth Lifestyle Program With Telephone Support (TXT2BFiT) to Prevent Unhealthy Weight Gain in Young Adults: Randomized Controlled Trial. JMIR mHealth uHealth. Canada; 2015;3(2):e66. PMID: 26076688.

49. Walthouwer MJL, Oenema A, Soetens K, Lechner L, De Vries H. Systematic development of a text-driven and a video-driven web-based computer-tailored obesity prevention intervention. BMC Public Health. England; 2013;13(1):978. PMID: 24138937.
